# BTX-A51 treatment modulates host innate immune responses via NF-κB and β-catenin signaling during Rift Valley fever virus infection *in vitro*

**DOI:** 10.1128/jvi.01042-26

**Published:** 2026-09-09

**Authors:** Rafaela Flor, Timothy Stocker, Kaylee Petraccione, Maryna Akhrymuk, Brittany N. Heath, Ivan Akhrymuk, Caitlin M. Woodson, Kylene Kehn-Hall

**Affiliations:** 1 Department of Biomedical Sciences and Pathobiology, Virginia Tech 1757, Blacksburg, Virginia, USA; 2 Center for Emerging, Zoonotic and Arthropod-borne Pathogens, Virginia Tech 1757, Blacksburg, Virginia, USA; University of Kentucky College of Medicine, Lexington, Kentucky, USA

**Keywords:** inflammation, NF-kb, CK1, Rift Valley fever virus, β-catenin

## Abstract

Rift Valley fever virus (RVFV) is a zoonotic and mosquito-borne hemorrhagic pathogen of high pandemic potential, endemic to most countries in Africa. It most severely causes disease in small domestic ruminants and is transmitted to humans. RVFV has been declared a priority pathogen by the World Health Organization for vaccine and therapeutic development. Outbreaks of this virus are known to cause great disturbances to the economy, agriculture, food availability, and healthcare system of local communities. No U.S. FDA-approved vaccines or therapeutics exist for the prevention or treatment of RVFV. Here, we used BTX-A51, an anticancer drug and casein kinase-1 (CK1) inhibitor, as a pharmacological tool to investigate the role of CK1 in regulating innate immune signaling during RVFV infection. The results show that CK1 inhibition via BTX-A51 dampened NF-κB-mediated signaling by blocking different steps in the cascade that require CK1 phosphorylation for signal progression. Consequently, this reduced translocation of p65 to the nucleus and transcriptional activation of pro-inflammatory cytokines and enzymes. Additionally, BTX-A51 treatment influenced the β-catenin pathway, resulting in a significant increase in β-catenin protein levels and β-catenin colocalization with p65. Delayed BTX-A51 treatment retained its impact on cytokine gene expression and partially rescued cells from RVFV-induced cell death. Our data suggest that CK1 inhibition influences both RVFV replication and host innate immune signaling, suggesting a relationship between the NF-κB and β-catenin pathways during RVFV infection. These findings identify CK1-mediated signaling as a potential target for host-directed therapeutic strategies aimed at limiting excessive virus-induced inflammation.

**IMPORTANCE:**

Rift Valley fever virus (RVFV) is a zoonotic and mosquito-borne pathogen of high pandemic risk, affecting ruminants and humans. Disease outbreaks cause great health and economic disruptions to communities across the African continent, being famously known for its “abortion storms.” Casein kinase 1 (CK1) is a host kinase ubiquitously present in eukaryotic cells and involved in multiple pathways, such as NF-κB-mediated inflammation and β-catenin signaling. Both NF-κB and β-catenin signaling are altered in RVFV-infected cells. This manuscript tests the ability of BTX-A51, a CK1 inhibitor, to limit viral replication and inflammatory signaling. BTX-A51 acted concomitantly on the NF-κB and β-catenin pathways to reduce proinflammatory signaling in RVFV-infected cells. Due to many mechanisms of infection being conserved across the Bunyavirales order, these findings also create the potential for BTX-A51 treatment to be tested against different viruses as a tool for pandemic preparedness.

## INTRODUCTION

Rift Valley fever virus (RVFV) is a mosquito-borne pathogen of significant concern to both veterinary and human health. The virus was originally described in Kenya in 1931 after a season of exceptionally high abortion rates and death in ewes and high mortality rates in lambs (1). Disease outbreaks are intimately related to heavy rainfall seasons and flooding, which facilitate the emergence of infected mosquitoes, most importantly of the Culex and Aedes genera (2). Mosquitoes are vectors and reservoirs of RVFV (3), transmitting the disease to its amplifying hosts: sheep, goats, and cattle (4). In newborn and young animals, the disease is acute and most severe, culminating in nearly a hundred percent mortality rate in some species, with signs of hemorrhagic fever, ocular disease, and liver disease (5). In older animals, presentation is often less severe, but outbreaks cause sweeping abortions across herds, known as “abortion storms,” commonly the first signs of Rift Valley fever, compromising animal reproductive health (2). Disease transmission also occurs via aerosolization of the virus present in infected blood and tissues, posing a risk to other animals and humans (6). In humans, symptoms are most commonly non-specific, flu-like, and self-resolving after 3–4 days, with mortality rates being less than one percent. In rare cases, however, the disease presents more severely, with hemorrhagic fever, jaundice, ocular disease, blindness, neurologic signs, and encephalitis, culminating in a fatal outcome (4). A case of vertical transmission in a pregnant Sudanese woman was reported in 2008 (7), and in a larger study in Sudan in 2016, 54% of RVFV-infected women experienced miscarriages (8).

As a neglected zoonotic disease, RVFV has gained increased attention due to its widespread presence in the African continent, in addition to its concerning expansion to the Middle East in 2000 (9). RVFV has been included in the World Health Organization’s R&D Blueprint for Epidemics (10) and categorized as a disease of high Public Health Emergency of International Concern (PHEIC) risk. Additionally, the World Health Organization (WHO) also characterized RVFV as a prototype pathogen, a virus representative of the Phenuiviridae family with high pandemic potential, whose resources should be prioritized and adaptable to other emerging threats (11). The absence of approved therapeutics and widely available vaccination for both humans and animals (12) emphasizes the urgency for continued effort to develop effective countermeasures against RVFV.

RVFV belongs to the Phenuiviridae family and Phlebovirus genus (13). It is a single-stranded negative-sense RNA virus with a tripartite genome, separated into large (L), medium (M), and small (S) segments that encode seven proteins (14). The L segment encodes the RNA-dependent RNA polymerase (RdRp), also called the L protein, responsible for cap-snatching, viral transcription, and replication (6). The M segment produces the envelope glycoproteins Gn and Gc that perform packaging and viral release, as well as the non-structural protein (NSm) and the 78-kDa protein (P78) (14, 15). The S segment utilizes an ambisense strategy to generate the structural nucleoprotein (N) and the non-structural protein NSs, the latter being the major virulence factor (16). The N protein is responsible for encapsidating the genomic RNA and forming the ribonucleoprotein complex (RNP) along with the L protein (14). The RVFV viral cycle takes approximately 10–12 h to complete once fusion in the cell’s plasma membrane takes place (14). Following entry, the viral RNPs are released into the cell, immediately undergoing transcription by the L-protein to create two types of positive-sense RNA strands: (i) mRNAs containing a 5′ cap (snatched from the host) for subsequent translation of viral proteins using cell machinery and (ii) antigenomic RNA that uses a *de novo* strategy to generate more copies to be packaged into new virions (6). The antigenomic S segment RNA is also used as a template to create the NSs mRNA. Assembly of the virus takes place in the Golgi complex, from where the envelope is formed, and the virus is eventually released via exocytosis (17).

Previous work by our group identified the host protein casein kinase-1 (CK1) as responsible for phosphorylating the RVFV L protein (18, 19). CK1 is a serine/threonine kinase ubiquitously expressed in all human tissues at different levels, participating in many different cell regulatory pathways, such as the Wnt/β-catenin pathway, hedgehog signaling, autophagy, NF-κB signaling, cell cycle regulation, as well as neurodegenerative diseases and cancers (20). Inhibition of CK1 via the small molecule inhibitor D4476 showed decreased viral replication of both RVFV MP12 and ZH501 strains in multiple cell types, in addition to decreased L protein phosphorylation compared to a DMSO control. These findings demonstrated the importance of CK1 in RVFV replication and its potential as a host-directed antiviral treatment, encouraging further investigation.

It has been proposed that the dysregulated inflammatory response promoted by RVFV infection is one of the main causes for significant loss of liver function, progression to neurological disease, and worsened patient prognosis (21–23). NF-κB signaling, a critical regulatory pathway of innate immune responses and inflammation, is induced in RVFV-infected cells, leading to the production of inflammatory cytokines and chemokines and culminating in apoptosis (22, 24). Inhibition of the NF-κB-dependent inflammation via treatment with curcumin consequently also reduces RVFV replication (25). CK1α has been identified as a key component of the NF-κB signaling cascade, acting as a “dual regulator.” Via association with the CARMA1/CARD11, BCL10, and MALT1 proteins (CBM complex) in B cells, CK1 first increases cytokine production and lymphocyte proliferation and later phosphorylates and inactivates the complex, terminating NF-κB signaling (26). Additionally, in other cell types, CK1 directly phosphorylates the S316 residue of p65, a major component of the NF-κB pathway, resulting in reduced p65 nuclear localization and transcriptional activity (27).

Given the importance of inflammation to RVFV disease progression and the interplay between CK1 and NF-κB signaling, our current work made use of BTX-A51, a small-molecule inhibitor targeting CK1α, as a pharmacological tool to investigate the role of CK1 in regulating host inflammatory signaling during RVFV infection. BTX-A51 was originally developed as an anti-cancer therapy and is currently in Phase II clinical trials for acute myeloid leukemia, metastatic breast cancer, and liposarcoma (28). Our results demonstrate that *in vitro* inhibition of CK1 in RVFV-infected cells via BTX-A51 resulted in decreased viral titers and viral RNA in multiple cell types, a reduction in gene expression and protein levels of components of the NF-κB pathway, and, consequently, a significant reduction in the gene expression of inflammatory cytokines, chemokines, and enzymes. Furthermore, CK1 inhibition caused significant changes in β-catenin signaling, suggesting a role in controlling excessive inflammation via modulation of both the β-catenin and NF-κB pathways. BTX-A51 also reduced interferon-β gene expression, which is a key limitation of its use. Despite this, treatment with BTX-A51 post-infection partially rescued cells from RVFV-induced cell death, providing a cell health benefit. Our data identify CK1-mediated signaling as a potential host-directed pathway to be further explored as an immunomodulatory strategy with the potential to protect cells from an excessive inflammatory state promoted by RVFV.

## RESULTS

### CK1 inhibition via BTX-A51 decreases RVFV replication in multiple cell types

Our previous work identified CK1 as a host kinase exploited by RVFV for the phosphorylation of its L protein (18). Additionally, CK1 inhibition via the well-characterized small-molecule inhibitor D4476 (29, 30) showed decreased viral replication of RVFV (18). However, although widely used for *in vitro* research, D4476 has not progressed in *in vivo* studies toward becoming a U.S. FDA-approved drug. For that reason and considering the promising data of CK1 inhibition as an antiviral therapy, a shift toward a new experimental drug, called BTX-A51, is explored in this manuscript. BTX-A51 is a multi-kinase inhibitor developed by BioTheryX as its lead clinical drug (31). The target for inhibition is primarily CK1 (more specifically CK1α) but also cyclin-dependent kinases 7 and 9 (CDK7 and CDK9) as an anticancer treatment against acute myeloid leukemia (AML) (32, 33).

**Fig 1:**
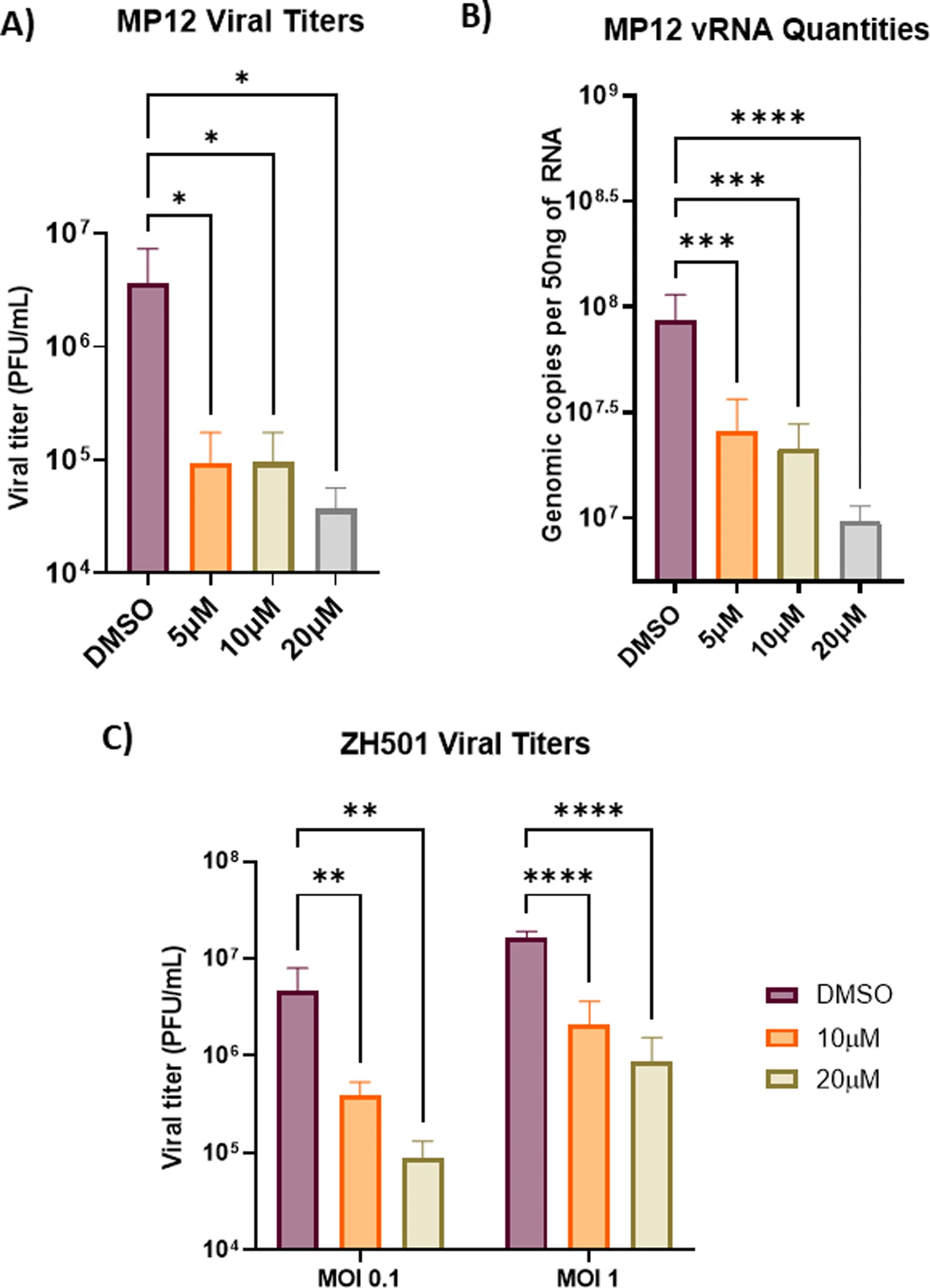
CK1 inhibition via BTX-A51 decreases RVFV replication. (**A and B**) HSAECs were pre-treated with DMSO control or BTX-A51 at increasing concentrations of 5, 10, and 20 µM for 1 h. Infection was performed with RVFV rMP-12 at an MOI of 0.1 for 1 h, and post-treatment was added back to cells after infection. At 24 hpi, the supernatants were collected for viral titers and lysates for viral RNA quantitation. (**C**) HSAEC cells were pre-treated with DMSO control or BTX-A51 at 10 and 20 µM, followed by RVFV ZH501 infection at an MOI of 0.1 or 1 for 1 h. Post-infection treatment was added back until the collection of supernatants at 24 hpi for viral titration. Data are shown as a mean ± SD (*n* = 4), with ordinary one-way ANOVA statistical analysis and Holm-Šídák’s (**A and B**) or Dunnett’s multiple comparisons test (**C**). Comparisons were performed between DMSO control-treated samples and BTX-A51-treated samples under different increasing concentrations. Only statistically significant comparisons are displayed. *P-value < 0.05, **P-value < 0.01, ***P-value < 0.001, ****P-value < 0.0001.

In order to determine the antiviral effect of BTX-A51, cytotoxicity experiments were first performed in human small airway epithelial cells (HSAECs) in the presence of BTX-A51 or a DMSO solvent control. Multiple concentrations from 5 to 640 µM were tested for 24 h, where BTX-A51 treatment at 5, 10, and 20 µM generated cell viability responses above 80% (Fig. S1A) and therefore was selected for further testing. Next, HSAECs were pre-treated for 1 h with BTX-A51 at 5, 10, and 20 µM, followed by a 1-h infection with either RVFV rMP12, a BSL-2 live-attenuated vaccine strain of RVFV, or ZH501, a pathogenic BSL-3 strain of RVFV, at a multiplicity of infection (MOI) of 0.1 or 1. Fresh BTX-A51 treatment was added back to cells in similar pre-treatment concentrations until the time of collection at 24 h post-infection (hpi). Supernatants were collected for viral titration (Fig. 1A and C), and in the case of rMP12-infected cells, the lysates were collected for viral RNA quantification via RT-qPCR (Fig. 1B). Treatment with BTX-A51 showed a significant decrease in viral titers and viral RNA when compared to the DMSO control, indicating that BTX-A51 has antiviral activity against RVFV.

**Fig 2:**
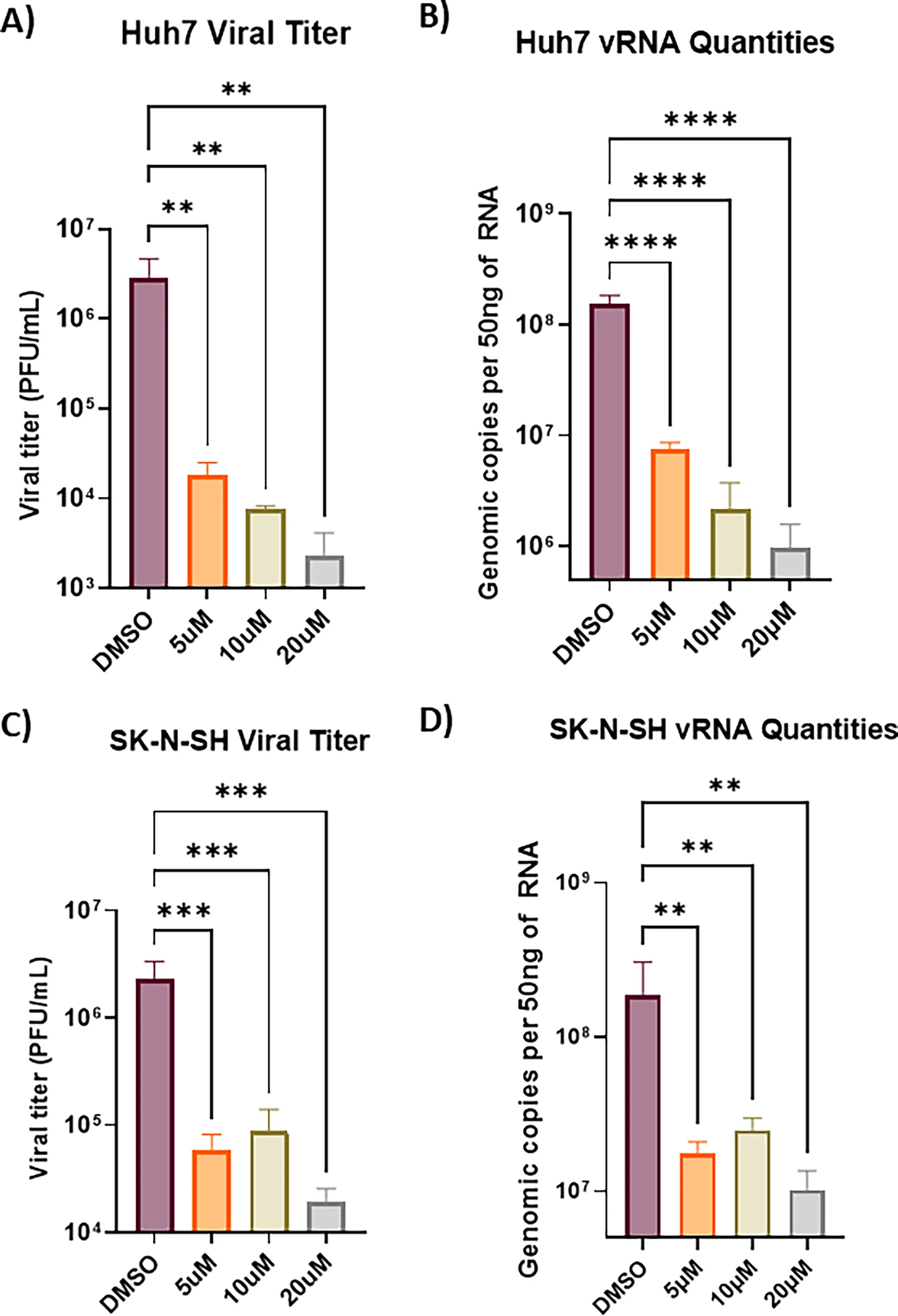
CK1 inhibition decreases RVFV replication in multiple cell types. Huh7 (**A and B**) and SK-N-SH cells (**C and D**) were pre-treated with DMSO control or BTX-A51 at increasing concentrations of 5, 10, and 20 µM for 1 h. Infection was performed with RVFV rMP-12 at an MOI of 0.1 for 1 h. Post-treatment was added back to the cells after infection. Supernatants were collected at 24 hpi for viral titers and lysates for viral RNA quantitation. Data are shown as a mean ± SD (*n* = 4), with ordinary one-way ANOVA statistical analysis and Holm-Šídák’s multiple comparisons test. Comparisons were performed between DMSO control-treated samples and BTX-A51-treated samples under different increasing concentrations. Only statistically significant comparisons are displayed. **P-value < 0.01, ***P-value < 0.001, ****P-value < 0.0001.

Based on RVFV pathogenesis and the most prominent pathologic features, other tissues of interest include the liver and the brain (4). To test whether BTX-A51 shows antiviral properties in the prominent cell types within the liver and brain, two additional cell lines were included: human hepatocellular carcinoma (Huh7) and human neuroblastoma (SK-N-SH) cells. Huh7 cells treated with BTX-A51 had ≥80% viability when treated with ≤20 µM of BTX-A51, and SK-N-SH cells had 100% viability when treated with ≤20 µM of BTX-A51 (Fig. S1B and C). Pre-treatment, RVFV rMP-12 infection (MOI 0.1), post-treatment, and 24 hpi collection were performed as described above. Cell supernatants were collected for infectious virus titration (Fig. 2A and C), and lysates were collected for intracellular viral RNA quantification (Fig. 2B and D). In these additional conditions, BTX-A51 showed similar, if not increased, antiviral effects compared to those observed in HSAECs, further validating its antiviral properties.

### BTX-A51 treatment inhibits NF-κB signaling

**Fig 3:**
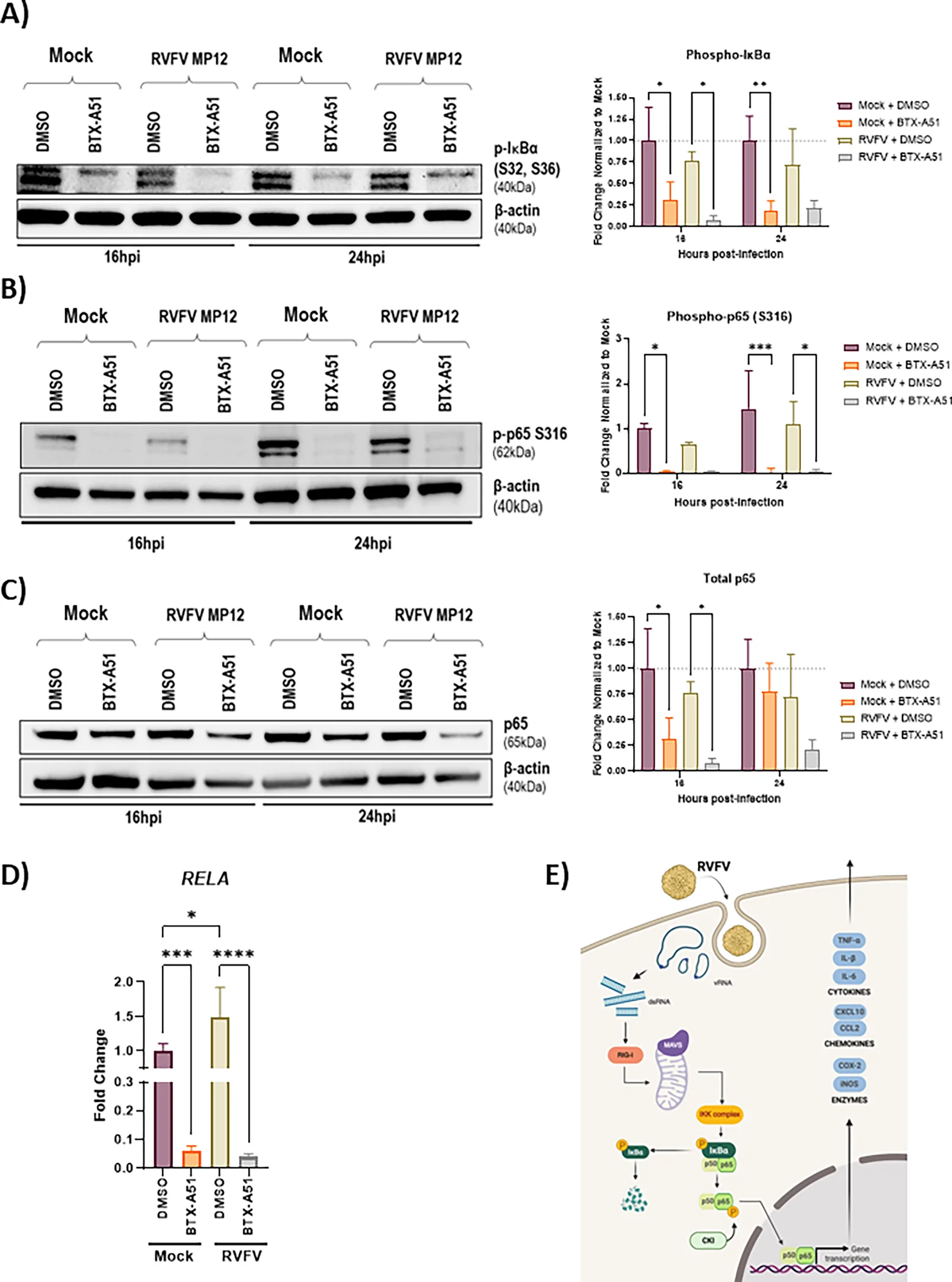
BTX-A51 treatment inhibits NF-κB signaling. HSAECs were treated with either a DMSO control or BTX-A51 at 10 µM for 1 h, followed by mock or RVFV rMP12 infection for 1 h. Post-infection treatment was added back to cells until the time points of collection (16 and 24 hpi). Protein lysates were analyzed via western blotting in a total of three replicates. Representative western blot images for the following proteins: (**A**) phospho-IκBα S32 and S36, (**B**) phospho-p65 S316, and (**C**) p65. ImageJ software was utilized for the relative quantification of each protein, using β-actin protein levels as loading controls for data normalization. (**D**) A separate set of samples was collected in RLT buffer for RNA extraction and p65 (RelA) gene expression quantitation via RT-qPCR. Data are shown as a mean ± SD (*n* = 3, panels A, B, and C; *n* = 4, panel D). Statistical analyses were performed using two-way ANOVA with Holm-Šídák’s multiple comparisons test. The following comparisons were assessed: Mock DMSO vs Mock BTX-A51, RVFV DMSO vs RVFV BTX-A51, and Mock DMSO vs RVFV DMSO. Only statistically significant comparisons are displayed. *P-value < 0.05, **P-value < 0.01, ***P-value < 0.001, ****P-value < 0.0001. (**E**) Graphic representation of NF-κB pathway activation upon RVFV infection. Created in BioRender. Kehn-Hall, K. (2026) https://BioRender.com/ons98t3.

CK1 directly impacts the NF-κB signaling cascade, phosphorylating NF-κB p65, resulting in reduced p65 nuclear localization and transcriptional activity (20, 27) (Fig. 3E). To investigate the impact of BTX-A51 on NF-κB signaling, key regulator events in the signaling pathway were determined via western blot analysis. HSAECs were pre-treated for 1 h with either DMSO (control) or BTX-A51 at 10 µM, followed by RVFV rMP-12 infection at an MOI of 0.1, with freshly prepared drug treatment at the same concentration added back to cells until the time of collection. A low multiplicity of infection was chosen in order to mimic slower-progressing natural infections. Cell lysates were collected at 16 and 24 hpi. An important indicator of NF-κB pathway activation is the phosphorylation of the inhibitor of nuclear factor kappa-B-α (IκBα). This protein is present in complex with p65 and p50 (part of the canonical arm of the pathway) in the cytoplasm of non-stimulated cells (34). Upon viral stimulation, the triggering of the cascade causes phosphorylation and ubiquitination of IκBα, marking it for proteasomal degradation. Once free, p65 translocates to the nucleus when it becomes transcriptionally active, causing the production of inflammatory cytokines and chemokines (35) (Fig. 3E). A significant decrease in phosphorylation of IκBα (serine [S] 32/S36) in mock and infected cells treated with BTX-A51 at 16 hpi was observed, with a similar trend at 24 hpi (Fig. 3A). These results indicate that BTX-A51 treatment inhibited IκBα phosphorylation.

In order to investigate a more targeted role of CK1 in NF-κB signaling, the impact of BTX-A51 on p65 S316 phosphorylation was investigated. p65 serine 316 is uniquely phosphorylated by CK1, which modulates its downstream effects, including its translocation into the nucleus and regulation of selected NF-κB-responsive genes (27). A dramatic decrease in phosphorylation of p65 at the S316 residue upon treatment with BTX-A51 was observed in both mock- and RVFV-infected cells (Fig. 3B). The overall p65 levels were also investigated. p65 protein levels were significantly decreased in both mock- and RVFV-infected cells treated with BTX-A51 at 16 hpi, with a similar trend at 24 hpi (Fig. 3C). The RelA gene, responsible for encoding the p65 protein, possesses several κB binding sites for all components of the NF-κB pathway, including p65 itself, indicating transactivation activity (36), prompting us to determine whether BTX-A51 was impacting p65 transcription. Considering this “positive feed-forward autoregulation” ability, it is possible that the decrease in p65 expression seen in BTX-A51-treated cells poses an impediment to the dysregulated activation of the pathway by its own protein, consequently controlling inflammation. Indeed, a significant reduction in p65 gene expression in BTX-A51-treated samples in comparison to a DMSO control was observed at 24 hpi (Fig. 3D), further demonstrating a decrease in the NF-κB signaling cascade.

### BTX-A51 treatment decreases NF-κB nuclear localization

**Fig 4:**
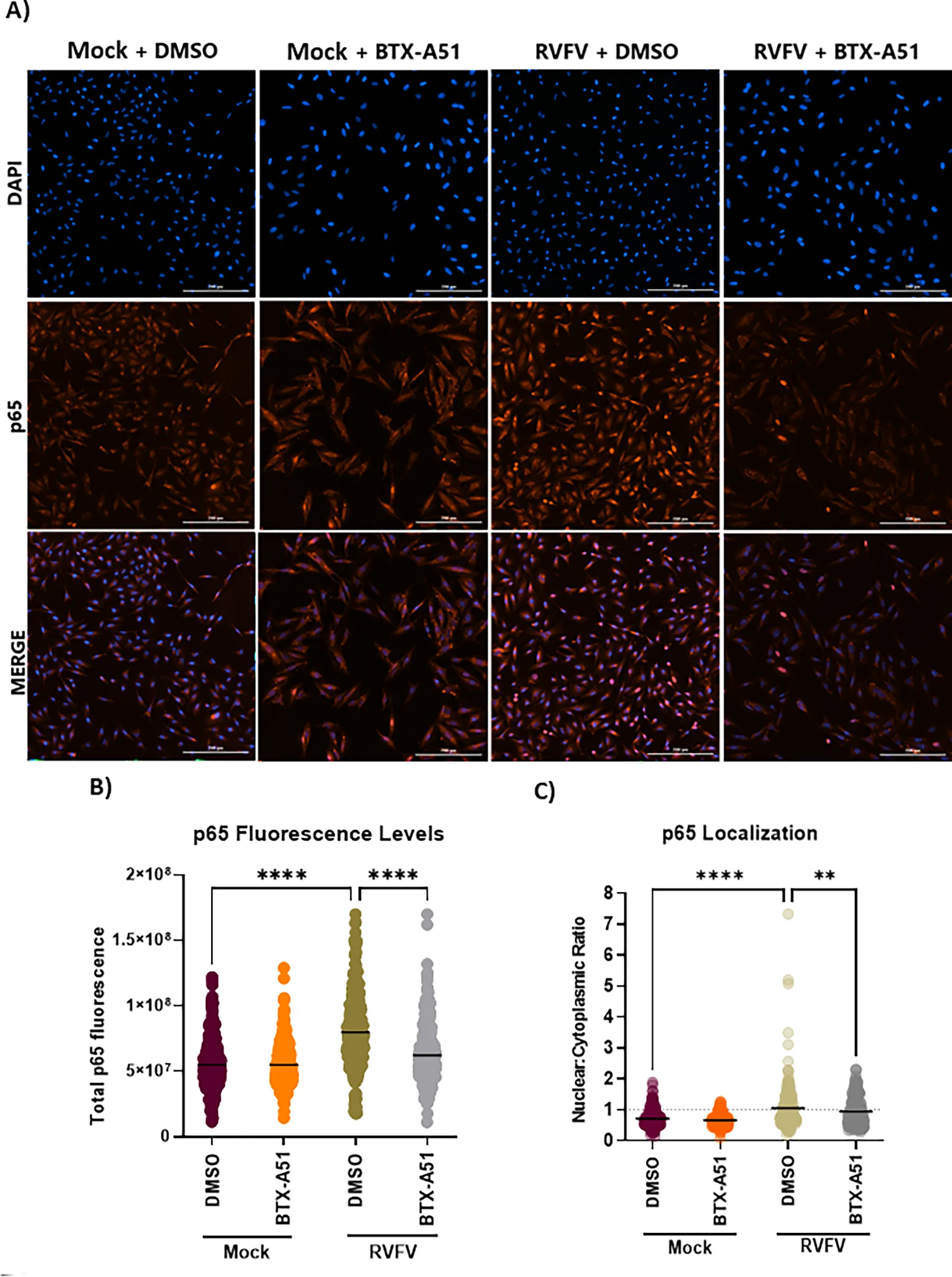
BTX-A51 treatment decreases NF-κB nuclear localization. (**A**) HSAECs were seeded on glass coverslips, pre-treated for 1 h with BTX-A51 (10 µM) or DMSO, infected with RVFV rMP12 (MOI 1), and post-treated. At 24 hpi, the cells were fixed, immunofluorescence staining was performed, and images were acquired via Cytation5. (**B and C**) Total p65 fluorescence intensity and cell location ratio were obtained for 270 cells via Cytation5 software and analyzed using GraphPad Prism. Data are shown as a mean ± SD (*n* = 270 cells). Statistical analyses were performed using two-way ANOVA with Holm-Šídák’s multiple comparisons test. The following comparisons were assessed: Mock DMSO vs Mock BTX-A51, RVFV DMSO vs RVFV BTX-A51, and Mock DMSO vs RVFV DMSO. Only statistically significant comparisons are displayed. **P-value < 0.01, ****P-value < 0.0001.

Following p65 phosphorylation, the protein must be translocated into the nucleus in order to stimulate NF-κB-dependent gene expression (34). Therefore, to further observe the effects of CK1 inhibition on the pathway’s signaling in RVFV-infected cells, immunofluorescence imaging was performed via widefield fluorescence microscopy using a Cytation5 plate reader. For this experiment, HSAECs were mock- or RVFV rMP-12-infected (MOI 1) and treated with either DMSO or BTX-A51 (10 µM). Immunofluorescence imaging (Fig. 4A) showed that in mock samples, treatment with BTX-A51 did not significantly impact overall p65 fluorescence intensity (quantified in Fig. 4B). However, upon RVFV infection, DMSO-treated cells had increased p65 levels, which were decreased in the presence of BTX-A51. These data are consistent with the analysis in Fig. 3C, where lower total p65 levels were observed in BTX-A51-treated cells. Additionally, p65 localization in the cells was assessed (Fig. 4C), where a <1 nuclear:cytoplasmic ratio indicates a higher cytoplasmic presence, while a >1 nuclear:cytoplasmic ratio indicates predominantly nuclear localization. The nuclear:cytoplasmic ratio between mock DMSO- and BTX-A51-treated cells was not significantly different. However, upon RVFV infection, DMSO-treated cells demonstrated more nuclear p65 in comparison to uninfected cells, demonstrating NF-κB pathway activation triggered by infection. In contrast, BTX-A51 treatment of infected cells had significantly more cytoplasmic localization of p65 when compared to DMSO, indicating that BTX-A51 reduced p65 nuclear localization. Collectively, these results coupled with data from Fig. 3 support the conclusion that BTX-A51 treatment inhibits NF-κB pathway activation in RVFV-infected cells.

### BTX-A51 treatment decreases NF-κB-dependent gene expression

To verify whether the impact of BTX-A51 treatment on the NF-κB cascade would translate into differences in NF-κB-dependent gene expression, a panel of cytokines, chemokines, enzymes, and interferons was analyzed via RT-qPCR. These groups of small inflammatory signaling proteins are extremely important for viral clearance and cell fate; however, an excess of these proteins may lead to cell death and further tissue damage. For this experiment, HSAECs were pre-treated for 1 h with either BTX-A51 (10 µM) or an equivalent volume of DMSO (control), mock- or RVFV rMP-12-infected (MOI 0.1) for 1 h, and subsequently post-treated with the respective treatment until collection at 24 h post-infection.

**Fig 5:**
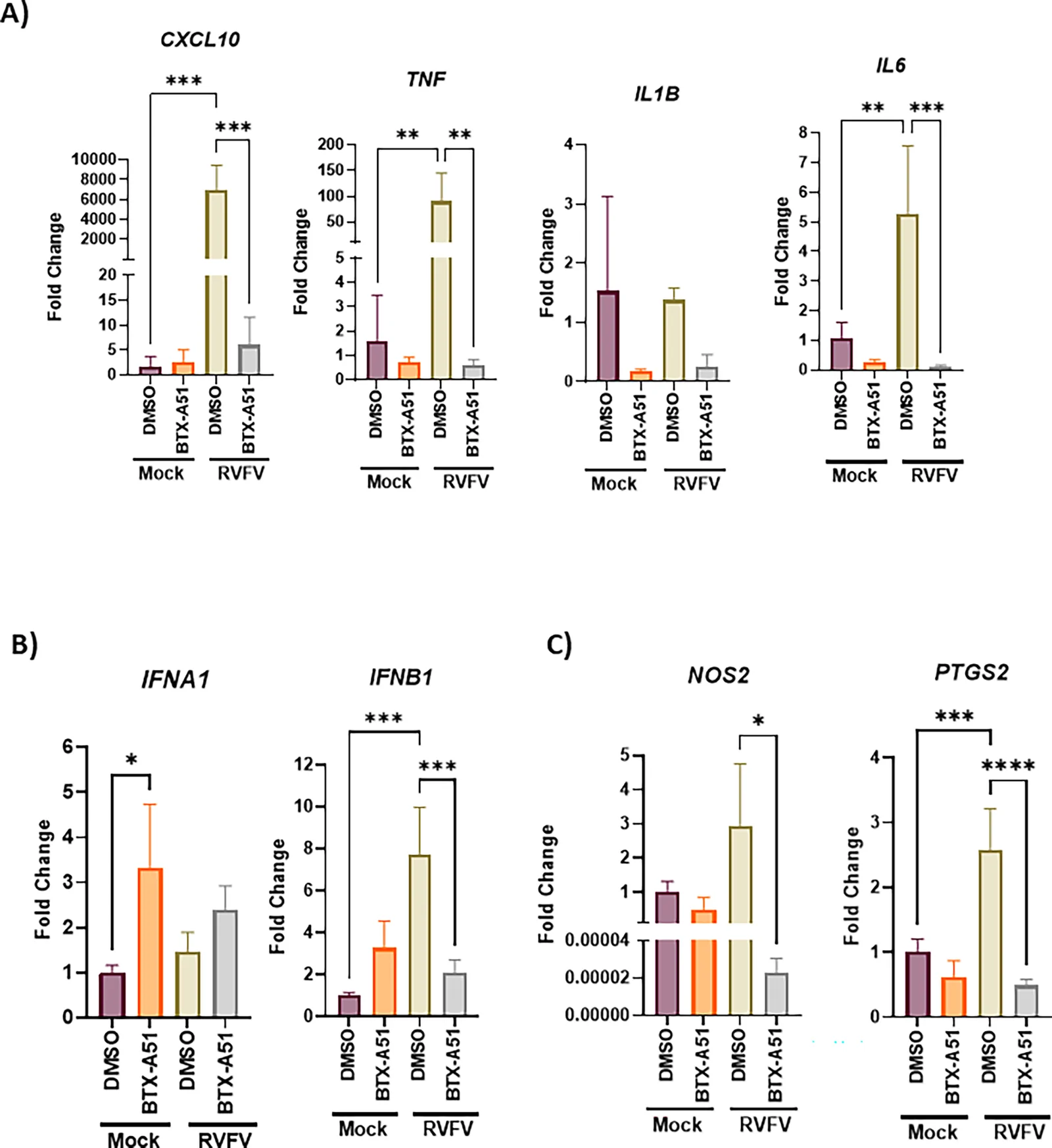
BTX-A51 treatment decreases NF-kB-dependent gene expression. HSAECs were pre-treated for 1 h with BTX-A51 (10 µM) or DMSO, infected with RVFV rMP12 (MOI 0.1), and post-treated until the 24 hpi time point. Cell lysates were collected in RLT buffer for gene expression analysis via RT-qPCR of the following genes: (**A**) cytokines CXCL10, TNF, IL1B, and IL6, (**B**) type I interferons α and β (IFNA1 and IFNB1), and (**C**) inflammatory enzymes NOS2 and PTGS2. Data are shown as a mean ± SD (*n* = 4). Statistical analyses were performed using two-way ANOVA with Holm-Šídák’s multiple comparisons test. The following comparisons were assessed: Mock DMSO vs Mock BTX-A51, RVFV DMSO vs RVFV BTX-A51, and Mock DMSO vs RVFV DMSO. Only statistically significant comparisons are displayed. **P-value < 0.01, ****P-value < 0.0001.

C-X-C motif chemokine ligand 10 (CXCL10), tumor necrosis factor-α (TNFA1), interleukin 1-β (IL1B), and interleukin 6 (IL6) encode inflammatory cytokines and possess κB-binding sites that are directly transactivated by p65 (37–40). Our results showed a sharp increase in gene expression of CXCL10, TNFA1, and IL6 upon RVFV infection, which is significantly reduced upon BTX-A51 treatment (Fig. 5A). Interestingly, IL1B was not increased in a comparison between mock and infected samples; however, in the presence of BTX-A51 treatment, its gene expression showed a reduced trend in both conditions.

Type I interferon genes are activated alongside the NF-κB pathway during infections upon cell sensing of pathogen-associated molecular patterns (PAMPs) by pattern recognition receptors (PRRs), and p65 is a component of the IFNB1 enhancesome, which further stimulates IFNB1 gene expression (41, 42). These two pathways are correlated and possess overlapping functions during antiviral responses (43). IFNA1 gene expression was significantly increased by BTX-A51 in mock-infected cells, but not in RVFV-infected cells nor following BTX-A51 treatment in RVFV-infected cells (Fig. 5B). IFNB1, on the other hand, was increased in RVFV-infected cells; however, a significant decrease was detected between BTX-A51 treatment and DMSO control during RVFV infection.

Enzymes such as inducible nitric oxide synthase (NOS2) and cyclooxygenase-2 (PTGS2) are directly transactivated by p65 (44, 45) and exert important functions of boosting the host’s defense mechanisms. NOS2 is responsible for inactivating viral proteins via nitrosylation and activating ciliary movement in the airways (46), whereas PTGS2 can assist with anti-inflammatory properties (47). However, prolonged activation of those enzymes can prove to be deleterious by causing oxidative stress and immune suppression (48). Both NOS2 and PTGS2 expression were elevated in RVFV-infected cells (Fig. 5C). A significant reduction in gene expression was observed for both genes in RVFV cells treated with BTX-A51, demonstrating an all-around effect of BTX-A51 in suppressing the NF-κB-dependent gene expression.

### BTX-A51 treatment decreases β-catenin phosphorylation and increases total β-catenin protein levels

**Fig 6:**
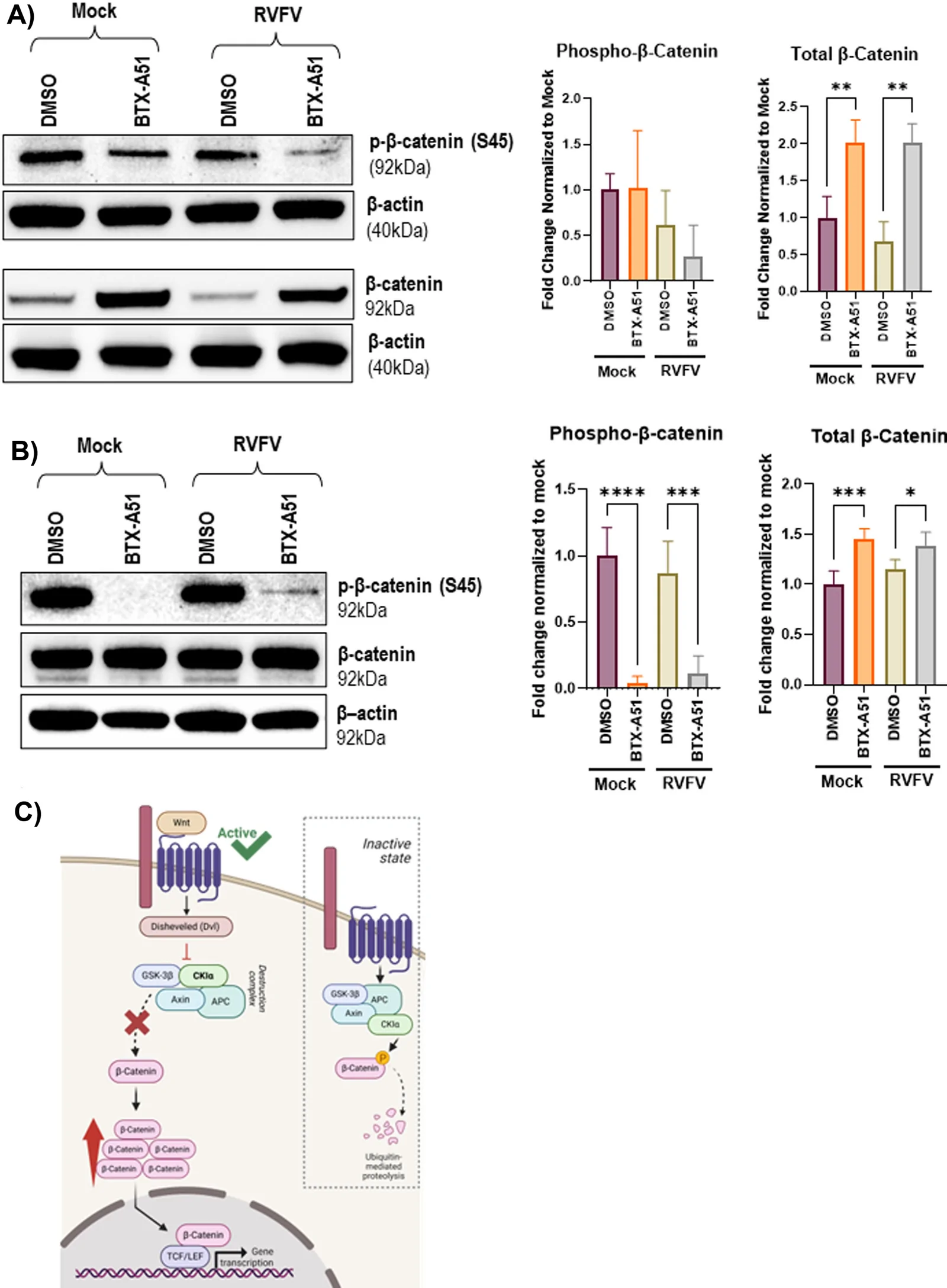
BTX-A51 treatment decreases β-catenin phosphorylation and increases total β-catenin protein levels. HSAECs (**A**) and Huh7s (**B**) were treated with DMSO control or BTX-A51 drug at 10 µM or 5 µM, respectively, for 1 h, followed by mock or RVFV rMP12 infection for 1 h. Post-infection treatment was added back to cells until the time point of collection (24 hpi). Protein lysates were analyzed via western blotting with representative images for phospho-β-catenin S45 and β-catenin. ImageJ software was utilized for the relative quantification of each protein, using β-actin as a loading control for data normalization. Data are shown as a mean ± SD (*n* = 3). Statistical analyses were performed using two-way ANOVA with Holm-Šídák’s multiple comparisons tests. The following comparisons were assessed: Mock DMSO vs Mock BTX-A51, RVFV DMSO vs RVFV BTX-A51, and Mock DMSO vs RVFV DMSO. Only statistically significant comparisons are displayed. *P-value < 0.05, **P-value < 0.01, ***P-value < 0.001, ****P-value < 0.0001. (**C**) Model of β-catenin metabolism. Created in BioRender. Kehn-Hall, K. (2026) https://BioRender.com/ons98t3.

Due to the effect of BTX-A51 as a CK1 inhibitor, it was of interest to investigate the possible effects on the Wnt/β-catenin pathway. This approach is justified by Wnt/β-catenin regulation via CK1 phosphorylation of β-catenin (S45), which marks it for proteasomal degradation (49) (Fig. 6C). Wnt/β-catenin signaling is important for homeostatic functions in cells, such as growth, proliferation, differentiation, and immune modulation (50). Most importantly to our studies, β-catenin has been identified as a key regulator of liver regeneration, an organ largely affected by RVFV infection (51). HSAECs and Huh7 cells were pre-treated with BTX-A51 for 1 h, followed by infection with RVFV rMP-12 at an MOI of 0.1, and BTX-A51 treatment was added back at the time points of collection. Cell lysates were collected at 24 hpi. Total β-catenin and phospho-β-catenin (S45) levels were determined via western blot analysis. A significant decrease in β-catenin phosphorylation was observed in cells treated with BTX-A51 in both HSAECs (Fig. 6A) and Huh7 (Fig. 6B) for mock- and RVFV-infected conditions. This suggests a decrease in its degradation and potential accumulation in the cell. We also analyzed the total β-catenin expression in both cell types, confirming its higher levels in BTX-A51-treated cells. In addition to CK1, GSK3-β sequentially phosphorylates β-catenin, leading to its degradation (52). GSK3-β activity is reflected by phosphorylation levels of its S9 residue, an inhibitory site. A significant decrease in GSK3-β S9 phosphorylation was observed in BTX-A51-treated cells (Fig. S2A and S2C). These data suggest that GSK3-β is inhibited, which is consistent with increased β-catenin levels upon BTX-A51 treatment (Fig. 6A and B). BTX-A51 treatment did not significantly impact GSK3-α phosphorylation (Fig. S2A and S2B), which is not involved in regulating the Wnt/β-catenin signaling cascade. Collectively, these data suggest that BTX-A51 treatment stimulated the Wnt/β-catenin signaling cascade through inhibiting β-catenin phosphorylation and increasing total β-catenin protein levels.

### BTX-A51-treated cells have increased β-catenin levels and cytoplasmic localization

**Fig 7:**
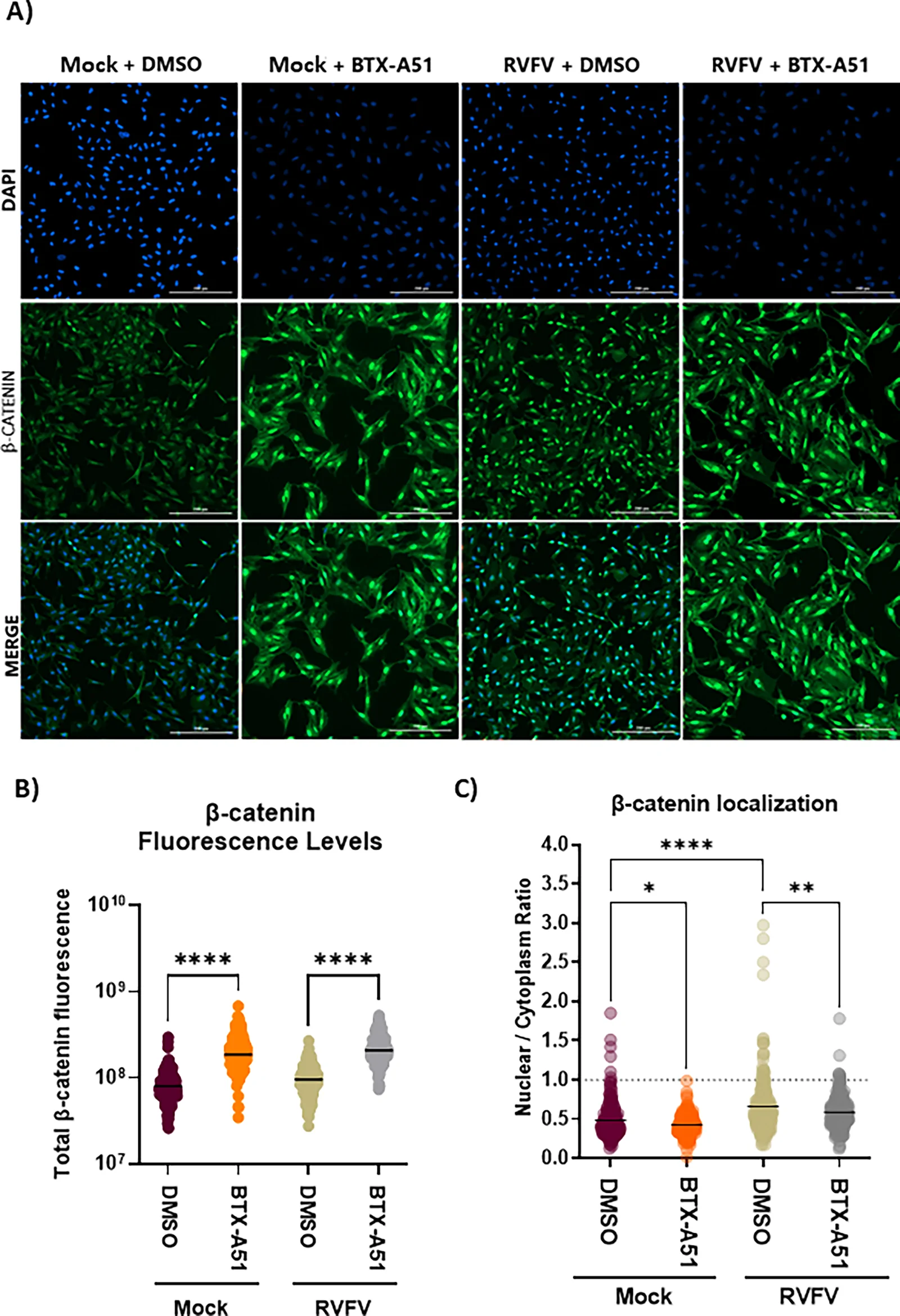
BTX-A51-treated cells have increased β-catenin cytoplasmic localization. (**A**) HSAECs were seeded on glass coverslips, pre-treated for 1 h with BTX-A51 (10 µM) or DMSO, infected with RVFV rMP12 (MOI 1), and post-treated. At 24 hpi, the cells were fixed and stained for DAPI (shown in blue) and β-catenin (shown in green) for immunofluorescence analysis via Cytation5. (**B and C**) Total β-catenin fluorescence intensity and cell location ratios were obtained via Cytation5 software and analyzed using GraphPad Prism. Data are shown as a mean ± SD (*n* = 235 cells). Statistical analyses were performed using two-way ANOVA with Holm-Šídák’s multiple comparisons tests. The following comparisons were assessed: Mock DMSO vs Mock BTX-A51, RVFV DMSO vs RVFV BTX-A51, and Mock DMSO vs RVFV DMSO. Only statistically significant comparisons are displayed. *P-value < 0.05, **P-value < 0.01, ****P-value < 0.0001.

An additional approach to study the effects of BTX-A51 on β-catenin was taken by observing potential changes in β-catenin localization in cells. Wnt/β-catenin pathway activation or the inhibition of β-catenin degradation leads to its accumulation in the cytoplasm and translocation to the nucleus, where it forms a complex with LEF/TCF (lymphoid enhancer factor/T-cell factor) and becomes transcriptionally active (53). To investigate this, HSAECs were seeded on glass coverslips for widefield immunofluorescence imaging. Pre-treatment was performed with DMSO or BTX-A51 at 10 µM for 1 h, followed by infection at an MOI of 1 with RVFV rMP-12 and post-treatment until the 24 hpi collection time. Our results show that upon BTX-A51 treatment, the total fluorescence signal for β-catenin increased (Fig. 7B). Its location was more nuclear in RVFV-infected and DMSO-treated cells (nuclear:cytoplasmic ratio >1) (Fig. 7C), suggesting the activation of the pathway. These results agree with previous literature that RVFV activates the Wnt/β-catenin pathway (54). However, upon treatment with BTX-A51, the β-catenin signal became more significantly cytoplasmic (nuclear:cytoplasmic ratio <1) (Fig. 7C), indicating that although β-catenin protein levels are increased, the corresponding increase in nuclear localization was not observed in RVFV-infected cells treated with BTX-A51.

### BTX-A51 treatment inhibits β-catenin-dependent gene expression

**Fig 8:**
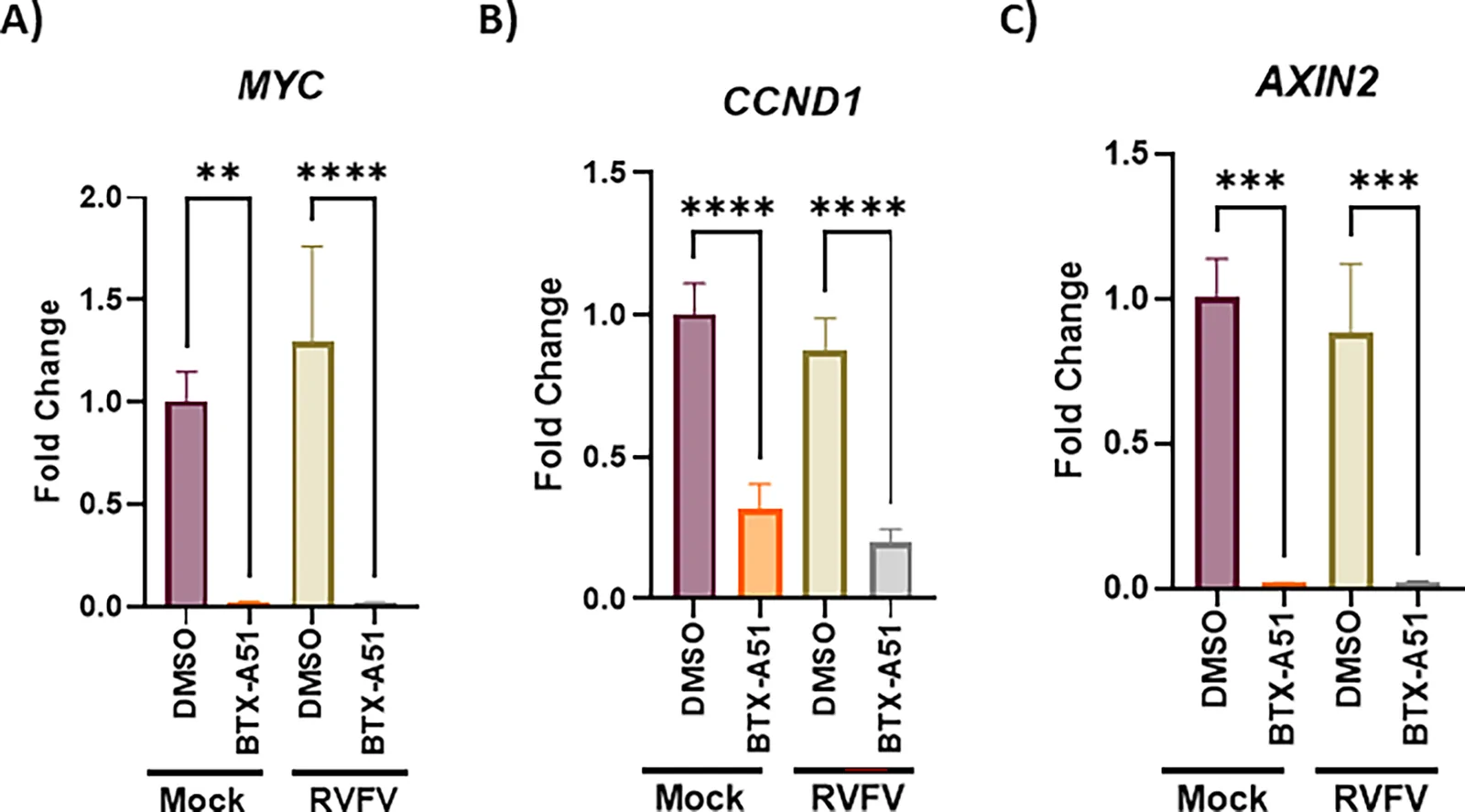
BTX-A51 treatment inhibits β-catenin-dependent gene expression. HSAECs were pre-treated for 1 h with BTX-A51 (10 µM) or DMSO, infected with RVFV rMP12 (MOI 0.1), and post-treated until 24 hpi. Cell lysates were collected in RLT buffer for gene expression analysis via RT-qPCR of the following β-catenin-dependent genes: (**A**) MYC, (**B**) CCND1, and (**C**) AXIN2. Data are shown as a mean ± SD (*n* = 4). Statistical analyses were performed using two-way ANOVA with Holm-Šídák’s multiple comparisons tests. The following comparisons were assessed: Mock DMSO vs Mock BTX-A51, RVFV DMSO vs RVFV BTX-A51, and Mock DMSO vs RVFV DMSO. Only statistically significant comparisons are displayed. **P-value < 0.01, ***P-value < 0.001, ****P-value < 0.0001.

Experiments were next performed to determine whether accumulation of and location changes in β-catenin in BTX-A51-treated cells would translate to altered gene expression. When transcriptionally active, β-catenin binds to its partners in the nucleus, TCF/LEF, which guide it to specific DNA loci (55). Of all Wnt/β-catenin-dependent genes, MYC, cyclin D1 (CCND1), and axis inhibition protein 2 (AXIN2) are some of the best characterized (53) and therefore selected here for further investigation. HSAECs were incubated with either BTX-A51 (10 µM) or an equivalent volume of DMSO (control) for 1 h before mock or RVFV rMP-12 infection (MOI 0.1). Following infection, the corresponding treatment was maintained until the time point of collection at 24 h post-infection. MYC, AXIN2, and CCND1 gene expression was sharply ablated in both mock and infected samples treated with BTX-A51 (Fig. 8A through C). These results are in agreement with data shown in Fig. 7, where although the β-catenin levels are higher in BTX-A51-treated cells, the signal is more significant in the cytoplasm than in the nucleus. These data indicate that BTX-A51 treatment suppresses β-catenin-dependent gene expression.

### p65 and β-catenin colocalize in BTX-A51-treated cells

**Fig 9:**
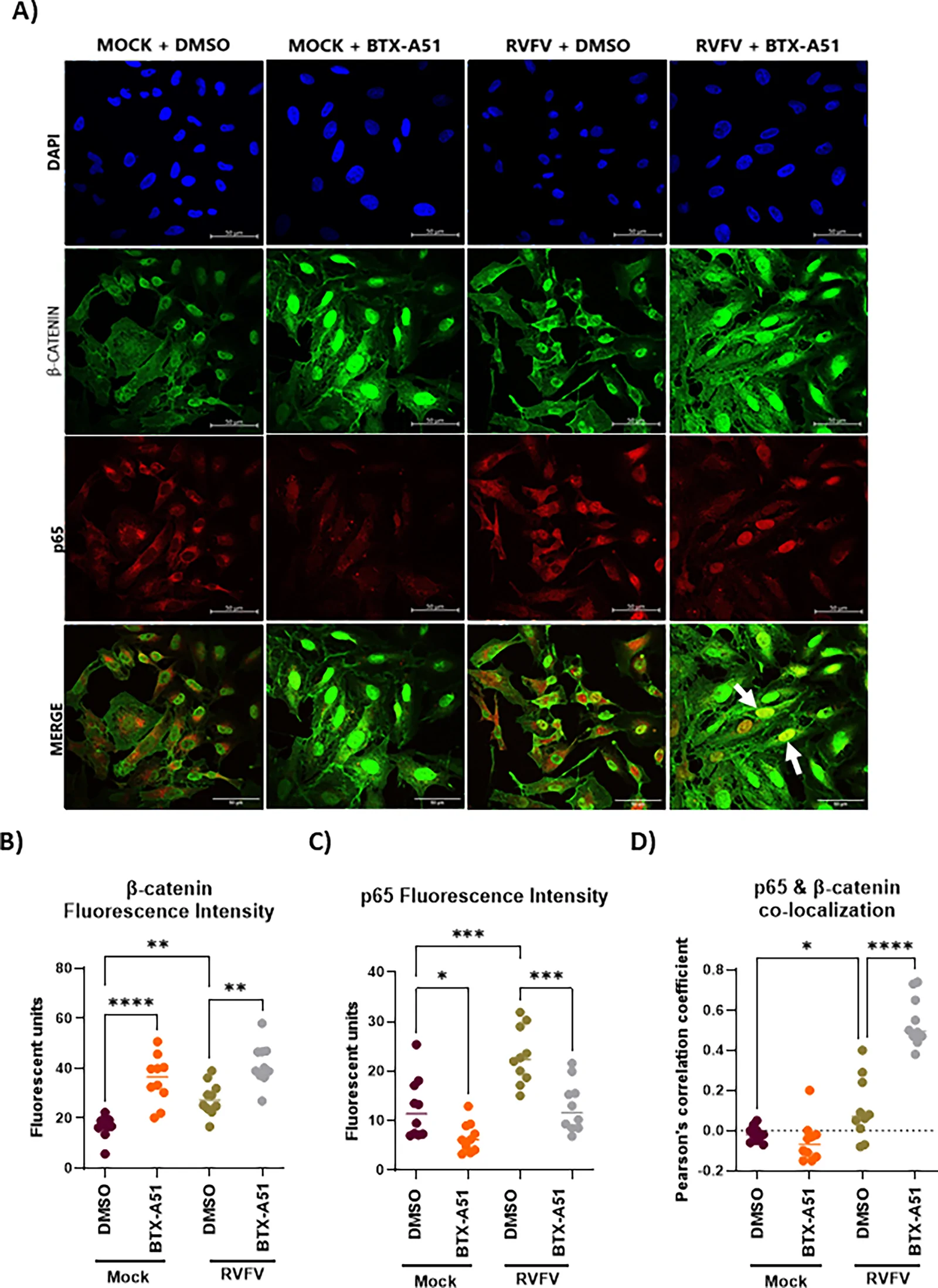
p65 and β-catenin colocalize in BTX-A51-treated cells. (**A**) HSAECs were seeded on glass coverslips, pre-treated for 1 h with BTX-A51 (10 µM) or DMSO, infected with RVFV rMP12 (MOI 1), and post-treated. At 24 hpi, the cells were fixed and stained for DAPI (shown in blue), β-catenin (shown in green), and p65 (shown in red) for confocal microscopy. White arrows represent two of the cells that were quantified. (**B and C**) Total β-catenin and p65 fluorescence intensity. (**D**) Pearson’s coefficient calculation for β-catenin and p65 cell colocalization. A total of 10 cells per condition were used for fluorescence intensity and Pearson’s coefficient calculations. Statistical analyses were performed using two-way ANOVA with Holm-Šídák’s multiple comparisons tests. The following comparisons were assessed: Mock DMSO vs Mock BTX-A51, RVFV DMSO vs RVFV BTX-A51, and Mock DMSO vs RVFV DMSO. Only statistically significant comparisons are displayed. *P-value < 0.05, **P-value < 0.01, ***P-value < 0.001, ****P-value < 0.0001.

Significant cross-talk between the NF-κB and β-catenin pathways has been identified over the years (56). One of the first studies to observe this interaction found that overexpression of β-catenin caused it to physically associate with both p50 and p65 components of the NF-κB pathway, generating interference in the ability of the proteins to bind to DNA, inhibiting NF-κB activity, and downstream gene expression (57). Furthermore, another study identified GSK-3β as responsible for phosphorylating the S468 residue of p65, which negatively regulates it (58). Our data thus far suggest that BTX-A51 treatment is responsible for the following: (i) β-catenin accumulation in the cytoplasm, (ii) a significant reduction in the overall p65 protein levels, and (iii) downregulation of NF-κB-dependent and β-catenin-dependent genes. Based on these results, we sought to determine if β-catenin and p65 are colocalizing in BTX-A51-treated cells. HSAECs were pre-treated for 1 h with either BTX-A51 (10 µM) or an equivalent volume of DMSO (control), mock- or RVFV rMP-12-infected for 1 h (MOI 1), and maintained under the same treatment conditions until collection at 24 h post-infection for confocal microscopy. As expected, based on our prior imaging studies (Fig. 4 and 7), increased signal intensity for β-catenin and decreased p65 signal were observed in BTX-A51-treated cells (Fig. 9A through C). p65 and β-catenin colocalization was readily observed in RVFV-infected cells treated with BTX-A51, especially in the nucleus (Fig. 9A). To support these results, the Pearson’s correlation coefficient (r) was determined. This coefficient identifies a co-localization relationship between two proteins that ranges between −1 and 1, where −1 means a negative correlation, +1, a positive correlation, and 0, no correlation. A significant increase in the Pearson’s coefficient was found in RVFV-infected cells treated with BTX-A51 (Fig. 9D), with r values ranging from 0.38 to 0.73, detecting co-localization between p65 and β-catenin. Meanwhile, RVFV-infected cells treated with DMSO showed an r value that ranged between −0.08 and 0.4, a significantly lower (*P* < 0.0001) co-localization correlation. This could be explained by the expected activation of the Wnt/β-catenin pathway in RVFV-infected cells (54). On the other hand, the more significant increase in p65 and β-catenin colocalization observed in BTX-A51-treated and -infected cells may indicate a potential interaction between β-catenin and p65 and, as seen in previous literature data (57, 59), suggest a model of suppression of NF-κB signaling via increased β-catenin levels. No colocalization was observed in mock-infected cells with or without BTX-A51 treatment.

### BTX-A51 treatment decreases NF-κB-dependent gene expression and reduces RVFV-induced cell death when administered as a post-infection treatment

**Fig 10:**
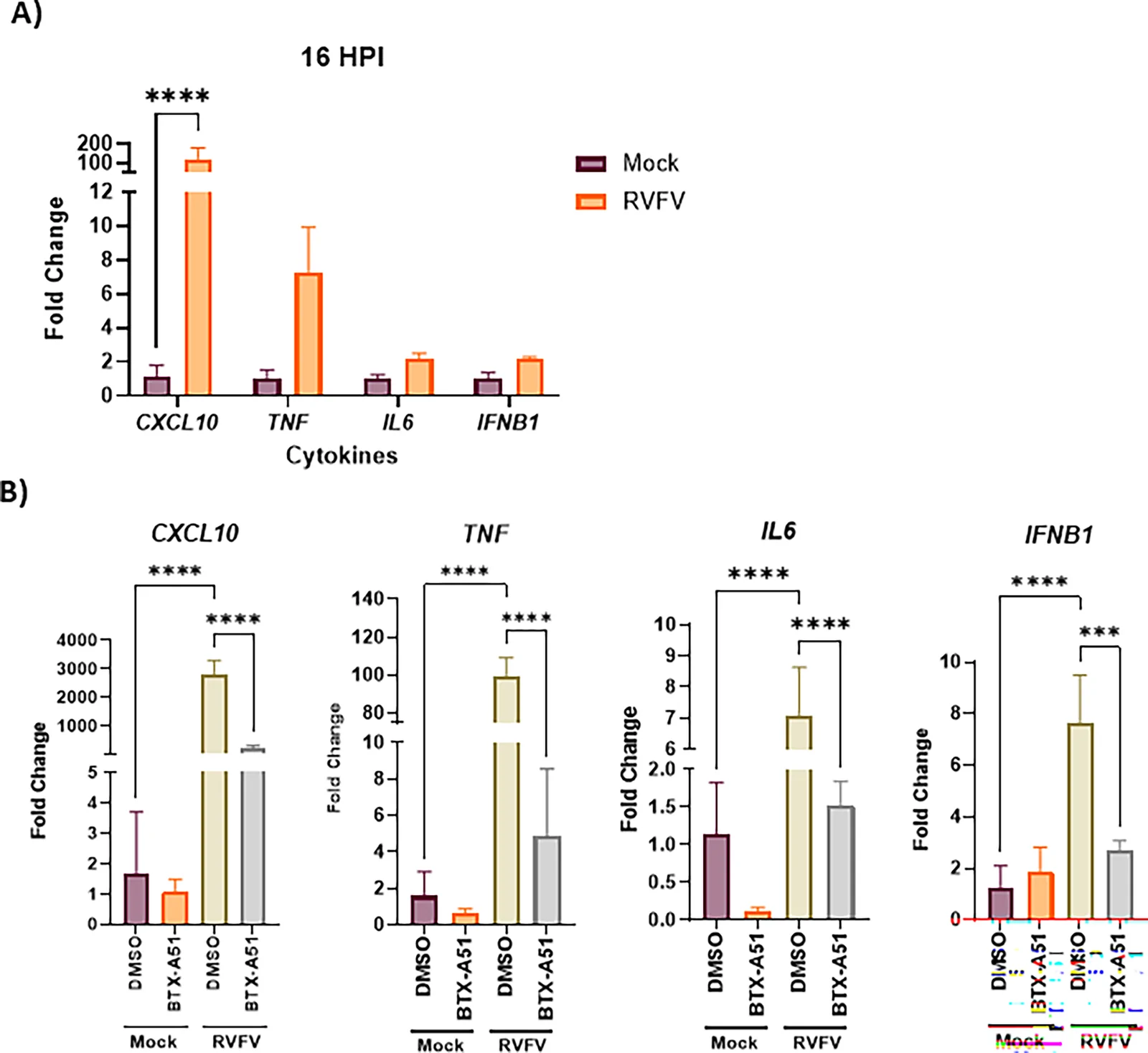
BTX-A51 modulates NF-κB-dependent gene expression when administered as a post-infection treatment. (**A**) HSAECs were mock- or RVFV rMP-12-infected at an MOI of 0.1 for 1 h, and cell lysates were collected at 16 hpi for RT-qPCR analysis of cytokine gene expression. Data are shown as a mean ± SD (*n* = 4). Statistical analyses were performed using two-way ANOVA with Holm-Šídák’s multiple comparisons tests (****P-value < 0.0001). Gene expression was analyzed for each cytokine by comparing mock- and RVFV-infected cells. (**B**) HSAECs were mock- or RVFV rMP-12-infected at an MOI of 0.1 for 1 h. No pre-treatment was performed; instead, cells received either DMSO (control) or BTX-A51 (10 μM) at 16 hpi. Cell lysates were collected at 24 hpi for RT-qPCR analysis of cytokine gene expression. Statistical analyses were performed using two-way ANOVA with Holm-Šídák’s multiple comparisons tests. The following comparisons were assessed: Mock DMSO vs Mock BTX-A51, RVFV DMSO vs RVFV BTX-A51, and Mock DMSO vs RVFV DMSO. Only statistically significant comparisons are displayed (***P-value < 0.001, ****P-value < 0.0001).

It is known that the NSs protein, as the major virulence factor of RVFV, causes a general shutdown of cell transcriptional events early during infection, thereby also affecting the host’s immune response to the virus (60). However, transcriptional activation of innate immune response genes has been documented in multiple experimental systems of RVFV infection (61–65), suggesting that the host is able to overcome the transcriptional blockage or that bystander uninfected cells are responding to infection. We hypothesized that delayed treatment of cells with BTX-A51 would enable an initial beneficial antiviral response to occur, followed by dampening of proinflammatory gene expression to provide an improvement in cellular health. Based on our previous work in HSAECs that showed robust innate immune gene expression occurring as early as 9 hpi (64), we opted to treat HSAECs with BTX-A51 at 16 hpi. To confirm that innate immune gene expression was increased prior to BTX-A51 treatment, we first assessed cytokine gene expression changes in untreated RVFV rMP-12-infected cells at 16 hpi. A significant increase in CXCL10 production, with increasing trends for TNF, IL-6, and IFNB1, was observed in RVFV rMP-12-infected cells compared to mock (Fig. 10A). Next, the cells were infected with RVFV rMP-12 (MOI 0.1), and BTX-A51 treatment was initiated at 16 hpi. The time point of collection remained at 24 hpi. RVFV-infected cells post-treated with DMSO displayed a significant increase in all cytokines tested compared to mock-infected cells post-treated with DMSO (Fig. 10B). In contrast, RVFV-infected cells post-treated with BTX-A51 had a significant reduction in cytokine gene expression when compared to RVFV-infected cells post-treated with DMSO (Fig. 10B).

**Fig 11:**
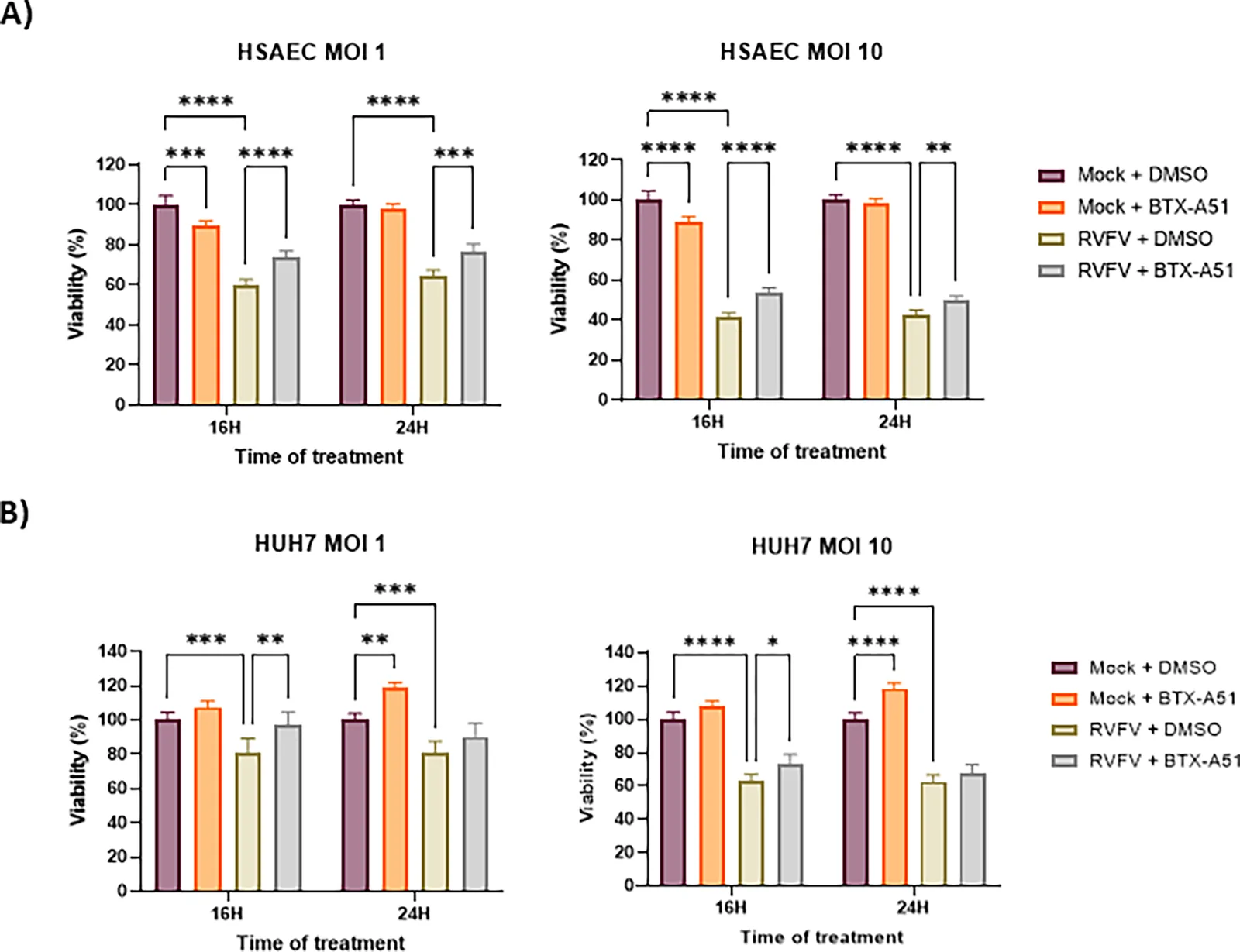
Post-infection BTX-A51 treatment improves cell viability following RVFV infection. HSAECs (**A**) and Huh7 cells (**B**) were mock- or RVFV rMP-12-infected at MOIs of 1 and 10 for 1 h. At 16 or 24 hpi, the cells were treated with either BTX-A51 (10 μM for HSAECs; 5 μM for Huh7 cells) or an equivalent volume of DMSO (control). Cell viability was assessed at 48 h post-infection. Data are shown as a mean ± SD (*n* = 4). Statistical analyses were performed using two-way ANOVA with Holm-Šídák’s multiple comparisons tests. The following comparisons were assessed: Mock DMSO vs Mock BTX-A51, RVFV DMSO vs RVFV BTX-A51, and Mock DMSO vs RVFV DMSO. Only statistically significant comparisons are displayed. *P-value < 0.05, **P-value < 0.01, ***P-value < 0.001, ****P-value < 0.0001.

To further determine the potential of BTX-A51 as a post-infection treatment, both HSAECs (Fig. 11A) and Huh7 cells (Fig. 11B) were either mock- or RVFV-infected (MOI of 1 and 10) and treated with either DMSO or BTX-A51 at 16 or 24 hpi. Cell viability was assessed at 48 hpi. Both cell types demonstrated significantly increased viability levels when cells were post-treated at 16 hpi, while HSAECs also showed significantly increased viability following treatment at 24 hpi. Together, these findings indicate that modulation of CK1-dependent signaling after infection is sufficient to improve cell viability during RVFV infection.

## DISCUSSION

Scientific efforts toward the treatment of viral infections have been present since the early 1960s through the discovery of directly acting antivirals (66). Unfortunately, the majority of those drugs became inefficient due to viral mutations, which resulted in resistance (67). A newer strategy to complement direct-acting antivirals has been devoted to the targeting of host proteins instead, which can be essential for the viral cycle and safely inhibited with much lower odds of resistance (67). Since the COVID-19 pandemic, the need for anti-inflammatory drugs and broad-spectrum antivirals of rapid availability has become evident (68). With that, a large movement toward the repurposing of drugs in the process of, or already FDA-approved, began. More recently, kinase inhibitors, often developed as anticancer and anti-inflammatory therapies, have been extensively tested for the treatment of viral infections (67). This approach stems from scientific evidence of the importance of multiple kinases for the viral cycle, as thoroughly reviewed by Hajjo et al. (67). Here, we evaluated BTX-A51, an orally bioavailable anticancer therapy that primarily targets casein kinase-1 (CK1), as a pharmacological tool to investigate the role of CK1 in RVFV infection and host p65-dependent inflammatory responses. CK1 was identified as a host kinase responsible for phosphorylating the L protein of RVFV (18). Considering the broad presence of CK1 in the cell, our focus was directed toward determining the impact of CK1 inhibition on inflammatory signaling via the NF-κB and β-catenin pathways, while exploring whether modulation of these pathways may reveal a potential host-directed therapeutic strategy.

**Fig 12:**
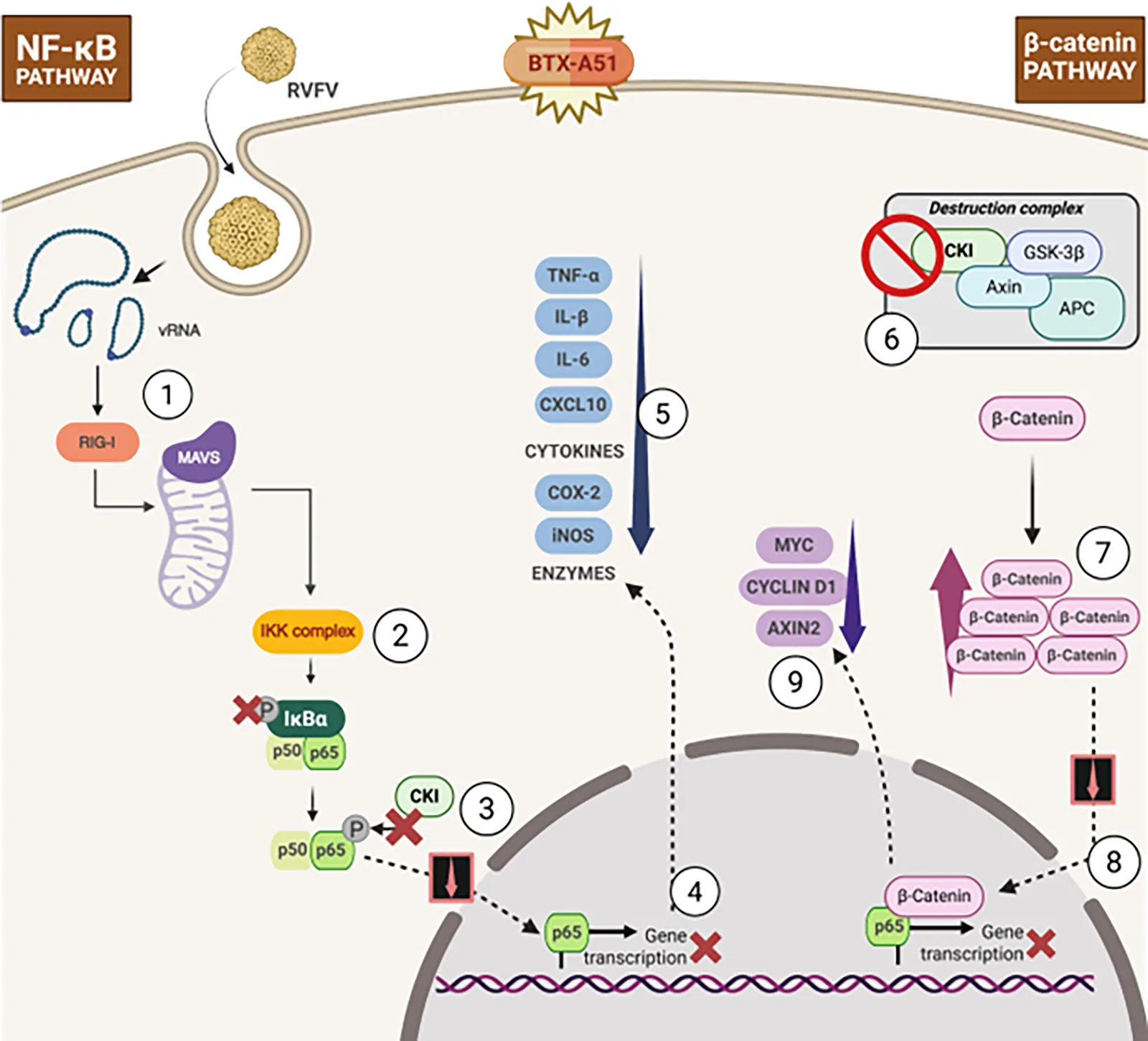
Working model of the impact of BTX-A51 on NF-kB and β-catenin signaling (1). Upon viral entry into the cell, RVFV releases its genome into the cytoplasm, which is detected by the retinoic acid-inducible gene I (RIG-I), a pattern recognition receptor (PRR), initiating the RIG-I/MAVS pathway (2). Subsequently, in DMSO-treated and RVFV-infected cells, RIG-I/MAVS causes activation of the IκB kinase (IKK) complex to phosphorylate the IκBα protein (an NF-κB pathway inhibitor), marking it for degradation. When phosphorylated, IκBα is degraded and frees p65 and p50 to enter the nucleus. However, upon BTX-A51 treatment, a significant decrease in IκBα phosphorylation occurs (3). Under DMSO treatment and RVFV infection, free p65 would suffer multiple post-translational modifications, including phosphorylation by CK1, that enable its nuclear translocation. However, BTX-A51 treatment suppressed p65 nuclear localization (4). With reduced nuclear translocation caused by BTX-A51 treatment, p65-dependent gene expression is reduced, including (5) inflammatory cytokines, chemokines, and enzymes involved in inflammatory processes (6). β-catenin levels in the cell are regulated by the “destruction complex” made up of CK1, GSK-3β, axin, and APC. CK1 is the first kinase to phosphorylate β-catenin, marking it for subsequent modifications by the other components until ubiquitin-proteasome degradation. However, upon CK1 inhibition via BTX-A51, the destruction complex cascade is not initiated, and β-catenin remains in the cytoplasm, causing its accumulation (7 and 8). The accumulation of β-catenin most commonly results in its translocation to the nucleus. Our working model shows that upon BTX-A51 treatment and RVFV infection, there is a reduction in β-catenin nuclear translocation when compared to DMSO treatment. Additionally, β-catenin was found to co-localize with p65 under those same conditions (9). Overall, the transcription of β-catenin-dependent genes was reduced upon BTX-A51 treatment. Created in BioRender. Kehn-hall, K. (2026) https://BioRender.com/ons98t3.

The NF-κB pathway is one of the main components of the innate and adaptive immune systems, triggering the production of pro-inflammatory cytokines and chemokines, and regulating inflammasomes (69). Many viruses across different families are known to manipulate the NF-κB pathway (70, 71). They modify different levels of the cascade to facilitate viral replication, prevent apoptosis, and modulate host immunity, helping evade it. CK1 is responsible for the phosphorylation of different proteins in the NF-κB pathway, such as components of the CARMA1/CARD11, BCL10, and MALT1 (CBM) complex (26), and of the p65 protein itself (20, 27). Our data showed that treatment with BTX-A51 reduced NF-κB-dependent gene expression through interference with multiple signaling events (Fig. 12). BTX-A51 treatment significantly decreased p65 S316 phosphorylation, which is a site exclusively phosphorylated by CK1 (Fig. 3B). It is known that p65 undergoes multiple post-translational modifications that, depending on the residues and their combinations, determine its ability to enter the nucleus, a pro- or anti-inflammatory activity, and the genes that become transcriptionally active (72). The absence of S316 phosphorylation was previously shown to reduce p65 nuclear localization (27), and in agreement with this, BTX-A51 treatment also reduced p65 nuclear localization in RVFV-infected cells (Fig. 4). Additionally, our results show that treatment with BTX-A51 caused a sharp reduction in phosphorylation of the IκBα inhibitory component (Fig. 3A), which suggests a decrease in its degradation and an increase in p65 retention in the cytoplasm. Our data suggest that these interferences to the pathway culminate in the sharp ablation of NF-κB-dependent cytokines and chemokines observed.

The Wnt/β-catenin pathway has both transcriptional and structural activity in the cell. In its transcriptionally inactive state, that is, in the absence of extracellular ligands, β-catenin is maintained at low levels—thanks to its destruction via a complex made up of CK1, GSK-3β, axin, and APC (50). In the presence of Wnt pathway ligands, however, this destruction complex is inhibited, and the β-catenin levels continuously rise, causing accumulation in the cytoplasm and subsequent translocation to the nucleus, inducing Wnt/β-catenin-dependent genes (53). Our results showed that BTX-A51 treatment caused a decrease in β-catenin S45 phosphorylation, a residue exclusively phosphorylated by CK1, leading to its accumulation (Fig. 6). Although nuclear translocation would be expected in this scenario, RVFV infection and BTX-A51 treatment caused its location to remain more significantly cytoplasmic, correlating with decreased β-catenin-dependent gene expression. Intricate interactions between the Wnt/β-catenin and NF-κB pathways have been well established, where, depending on the cell context and external stimuli, β-catenin is able to physically form a complex with both p65 and p50 proteins in the cytoplasm and/or in the nucleus and dampen the transcription of NF-κB-dependent genes (56). A study by Du et al. (59) showed via coimmunoprecipitation that β-catenin physically interacts with both the p65 and the p50 NF-κB subunits in primary human hepatocytes, HepG2 cells, and colon cancer cells, inversely correlating with cytokine-induced iNOS expression (59). They demonstrated that high levels of β-catenin inhibited NF-κB-mediated gene expression not only of iNOS but also of other NF-κB-dependent genes, Traf1 and Fas. As an added layer of verification of their hypothesis, epithelial lung cancer cells were treated with lithium chloride, an inhibitor of glycogen synthase kinase 3β (GSK-3β), causing accumulation of β-catenin and inhibiting TNFα-stimulated p65. A prior study by Deng et al. (57) obtained similar results, where β-catenin interacted and inhibited NF-κB-dependent signaling in human colon and breast cancer (57). They further emphasized how this regulatory effect of β-catenin is obtained via the physical interaction with p65 and is independent of T cell factor (TCF)-mediated transcription. Indeed, our data demonstrated a significant increase in β-catenin and p65 colocalization in RVFV-infected HSAECs treated with BTX-A51. Based on this, our working model is that in the condition of RVFV infection and treatment with BTX-A51, the role of β-catenin may shift to interfere with NF-κB-dependent signaling in a TCF-mediated transcription-independent manner.

In addition to targeting CK1, BTX-A51 is also known to inhibit the activity of CDK7 and CDK9 in acute myeloid leukemia models (28). These kinases are responsible for the phosphorylation of the C-terminal domain (CTD) of RNA polymerase II, participating in transcription initiation and elongation, especially of genes with super-enhancers (28). This effect must be taken into consideration for a potential overall decrease in gene transcription of treated cells. However, it should be noted that while most genes analyzed were downregulated following BTX-A51 treatment, expression of IFNA1 was unaffected or increased in treated cells. This suggests that a global suppression of gene expression is not occurring. 18S ribosomal RNA (rRNA) was used as our housekeeping gene, demonstrating no statistically significant difference in cycle threshold (Ct) values between all four conditions (Fig. S3). 18S RNA is transcribed by RNA polymerase I (73), which is canonically not impacted by CDK7 or CDK9.

CK1 is a critical regulator of the cell, and thus, a careful balance between the impacts on viral infection and cell viability must be considered. Upon treatment with BTX-A51, cell cultures displayed a modest reduction in cell viability (reduced ATP production), a finding not entirely unexpected, given the established role of CK1 in regulating cell cycle progression and proliferation (74–76). Because ATP-based viability assays do not distinguish between cytotoxicity and reduced cellular proliferation, these measurements may reflect a cytostatic effect of CK1 inhibition rather than cell death. Total cell counts were not significantly different in cells treated with BTX-A51 (Fig. S4), suggesting a cytostatic impact. Comparison of the antiviral efficacy data with the viability data suggests that the reduction in viral titers cannot be explained solely by the apparent decrease in viability. For example, Huh7 cells treated with 10 μM of BTX-A51 retained nearly 100% viability while exhibiting an approximately 2.5-log reduction in viral titers compared with DMSO-treated controls. Likewise, SK-N-SH cells maintained nearly 100% viability at 20 μM of BTX-A51 while demonstrating an approximately 1.5-log reduction in viral titers.

A limitation of our current work is that the impact of CK1 inhibition via BTX-A51 on RVFV pathogenesis and inflammation was not determined *in vivo*. In addition, the observed downregulation of IFNB1 represents a potential limitation of CK1 inhibition as a host-directed therapeutic strategy. Although modulation of inflammation may be beneficial, suppression of type I interferon responses could also reduce an important component of the host antiviral defenses. Future studies are necessary to identify the optimal dose of BTX-A51 for the treatment of RVFV-promoted inflammation, as well as the treatment window for maximum response efficiency. The correct timing for the administration of anti-inflammatory, immunomodulatory, and antiviral drugs upon infection is an important consideration and has been widely discussed in the literature. A study by Plaçais et al. (77) addressed the high efficiency discrepancies in the use of immunotherapies observed by physicians when treating severe COVID-19 patients. Other studies (78–81) also report the importance of timeliness when treating inflammation, where viral pathogenesis and cytokine storm onset must be considered for a successful outcome. Given that BTX-A51 treatment reduces interferon-β gene expression, early treatment of BTX-A51 would likely exacerbate disease. In addition, potential toxicity of BTX-A51 has to be carefully evaluated and may limit dosing options. Combining host-directed therapeutics with direct-acting antivirals is a potential approach to overcoming the limitations of BTX-A51. In support of this, a recent study found that treatment of RVFV-infected mice with nitazoxanide and the direct-acting antiviral favipiravir led to an increase in the time to death (82).

RVFV is one of the most well-studied viruses within the Bunyavirales order and is considered a prototype for the development of countermeasures (83). It is a hemorrhagic virus of pandemic and bioweapon potential, with no FDA-approved therapeutics for emergency use (83, 84). An effective immunomodulatory treatment with antiviral properties against RVFV, such as BTX-A51, could signify an important step toward pandemic preparedness for the entire Bunyavirales order. Altogether, our work has addressed the lack of RVFV countermeasures and provided supporting findings to suggest the repurposing of BTX-A51 to improve patient prognosis, while also unveiling important mechanistic aspects of the virus. In severe RVFV cases, the dysregulated inflammatory state promoted by the virus has been detected as one of the main causes of liver failure and neurological disease progression. Host-directed treatments, such as BTX-A51, targeting a kinase key to the inflammatory process specifically triggered by RVFV, allow for a more focused and efficient way to improve patient prognosis. Furthermore, given the role of CK1 in directly phosphorylating the virus’ RNA-dependent RNA polymerase (L protein) (18), its inhibition generates a moderate antiviral activity. This effect adds another advantage to the treatment approach. Our data demonstrated promising results regarding the implementation of BTX-A51 as a complementary anti-inflammatory treatment for viral infections.

## MATERIALS AND METHODS

### Cell culture

HSAECs were acquired from Cambrex Inc., Walkersville, MD, and cultivated in Ham’s F12 culture media (Corning, Cat No. 10-080-CV), enriched with 10% fetal bovine serum (FBS), 1% penicillin and streptomycin, 1% L-glutamine, 1% non-essential amino acids (NEAA), 1% sodium pyruvate, and 0.1% 1000 X 2-mercaptoethanol (Gibco, Cat No. 21985023). Media were filtered using a bottle-top vacuum filter system (Corning, Cat No. CLS431097). Vero cells (African monkey kidney) were obtained from American Type Culture Collection (ATCC, CCL-81) and maintained using Dulbecco’s modified Eagle’s medium (DMEM) (Corning, Cat No. MT10027CV; Gibco, Cat No. 11965092) supplemented with 10% FBS, 1% penicillin and streptomycin, and 1% L-glutamine. Hepatocyte-derived carcinoma cells (Huh7) were cultured in DMEM medium, enriched with 10% FBS. SK-N-SH cells (neuroblastoma cell line) were maintained in Eagle’s minimum essential medium (EMEM) supplemented with 10% FBS. All cell culture was passaged using phosphate-buffered saline (PBS) (Gibco, Cat No. 10010023) and Trypsin-EDTA (0.25%) phenol red (Gibco, Cat No. 25200056), and the cells were maintained at 37°C and 5% CO_2_.

### Virus infections

RVFV infections were performed at an MOI of 0.1 or 1, with inoculum preparation in cell growth medium to a final volume of 200 µL per well in 12-well plate vessels. Infections of cells in glass coverslips for immunofluorescence imaging were adapted to a 400 µL total volume in a six-well plate format. Infections were carried out in cell culture incubators under 37°C temperature and 5% CO2 conditions for 1 h, with gentle rocking of plates every 15 min to ensure equal inoculum distribution and avoid drying of the cell monolayer. Viruses utilized for experiments were recombinant MP12 (rMP12) strain derived from the virulent ZH501 virus (85), allowing for use under biosafety level 2 (BSL-2) requirements, and RVFV ZH501, provided by Dr. Stuart Nichol from the U.S. Centers for Disease Control and Prevention (CDC), under BSL-3 laboratory conditions following select agent regulations.

### Plaque assay

Quantification of infectious virus was performed via plaque assay of cellular supernatants, as previously described (86).

### Western blot analysis

Upon determined time-point collection, the cells were washed two times with phosphate-buffered saline (PBS) (Gibco, Cat No. 10010023) and lysed using blue lysis buffer (2× Novex Tris-Glycine SDS Sample Buffer [Thermo Fisher Scientific, Cat# LC2676], Tissue Protein Extraction Reagent [T-PER; Thermo Fisher Scientific, Cat# 78510], 2.0 mM EDTA pH 8.0 [Invitrogen, Cat No. 15575020], 0.8 mM NaF, 160 μM Na3VO4, 26 mM dithiothreitol [DTT], and EDTA-free Complete Protease Inhibitor Cocktail tablet [Sigma-Aldrich, Cat#11873580001]); 10% β-mercaptoethanol (Millipore Sigma, Cat. No. 444203-250ML) was added to blue lysis buffer at a final concentration of 10% immediately before sample collection. Lysed samples were boiled for 10 min at 90°C–100°C and loaded into a 4%–12% Bis-Tris glycine gel (Thermo Fisher Scientific, Cat No. NP0321) at a 20–25 µL volume. Gels were run using a Mini Gel Tank wet electrophoresis method (Thermo Fisher, Cat No. NW2000) for 35–45 min at a constant 180 V in 5% MOPS buffer (Invitrogen, Cat No. NP0001). Protein transfers to a polyvinylidene fluoride microporous film membrane (PVDV) (VWR, 490007) were performed using a Mini Gel Tank and module (Thermo Fisher Scientific, Cat No. NP0321) in transfer buffer (Tris-Glycine Buffer [Cat No.], methanol [VWR, Cat No. BDH1135-4LG], and ultrapure type 1 water) for 1 h at 4°C. Membranes were blocked at room temperature for 1 h using 5% bovine serum albumin (BSA) (Thermo Fisher, Cat No. B14) or milk in Tris-buffered saline solution containing 0.1% Tween-20 (TBST) (Promega, Cat No. H5151). Membranes were probed with antibodies diluted at 1:500 or 1:1,000 (antibody specific) in 5% BSA blocking buffer and incubated overnight at 4°C. Membranes were washed with Tris-buffered saline (TBS) and TBST in four 5-min washes for 20 min and incubated with secondary antibody for 1 h at room temperature. Secondary antibodies used were anti-rabbit (Invitrogen, Cat No. 32460) or anti-mouse (Invitrogen, Cat No. 32430) diluted at 1:1,000 in 5% BSA blocking buffer. Membrane washes were repeated as abovementioned, and imaged via chemiluminescence using SuperSignal West Femto Maximum Sensitivity substrate kit (Thermo Scientific, Cat No. 34095) in the Bio-Rad ChemiDoc MP Imaging System. Antibodies used: Anti-Casein Kinase 1-α antibody (Abcam, Cat No. EPR19824), Phospho-CK1-α (Thr321) (Invitrogen, Cat No. PA5-36790), β-catenin (Cell Signaling, Cat No. 9562S), phospho-β-catenin (Ser45) (Cell Signaling, Cat No. 9564S), NF-κB p65 (Cell Signaling, Cat No. 8242S), phospho-NF-kB p65 (Ser316) (Antibodies Inc., Cat No. p1350-316), phospho-IkB-α (Ser32 and Ser36) (Invitrogen, Cat No. 137401), phospho-GSK-3α/β (Ser21/9) (Cell Signaling, Cat No. 9331S), and HRP-conjugated β-actin (Abcam, Cat#ab49900). Relative protein levels were quantified via ImageJ software, using β-actin as a loading control for normalization.

### Inhibitor treatments

BTX-A51 or Casein Kinase Inhibitor 51, a small molecule inhibitor, was obtained from MedChemExpress (Cat No. HY-123954) in solid form. Upon arrival, the drug was resuspended in dimethyl sulfoxide (DMSO) (ATCC, Cat No. 4-X-5) to a final concentration of 10 mM, aliquoted, and stored at −80°C for a maximum time of 6 months, according to the manufacturer’s instructions. Drug treatments were performed in concentrations of 5, 10, and 20 µM of BTX-A51 diluted in complete cell medium. Control treatments were performed using a corresponding volume of DMSO. Cells were seeded 24 h prior to the experiment start at 1.5 × 10^5^ cells per well in 12-well plates. On the day of the experiment, existing media were removed via aspiration, and 1 mL of fresh complete medium containing BTX-A51 or DMSO was added to wells for 1 h. Cells were kept in an incubator at 37°C and 5% CO2, determining the pre-treatment period. After 1-h pre-treatment, cells were infected with RVFV rMP12 or ZH501 virus at MOI 0.1 or 1 at a final volume of 200 µL for 1 h, being gently rocked every 15 min to ensure even distribution of inoculum and keep the cell monolayer from drying. After infection, the cells were washed twice with PBS, and complete medium containing freshly diluted drug or DMSO was added back to the cells until the time point of collection.

### CellTiter-Glo

HSAEC, Huh7, and SK-N-SH cells were seeded in white-wall 96-well plates at 2 × 10^4^ cells/well. BTX-A51 and DMSO serial dilutions were performed starting from 40 µM and carried out to 0.3125 µM, and the resulting dilutions were added to the corresponding wells containing seeded cells. Drug treatments were left for 24 h. Twenty-four hours post-treatment, existing treatment was removed from wells, and 100 µL of fresh media was added back, along with 100 µL of CellTiter-Glo reagent (Promega, Cat No. G7571), as per the manufacturer’s instructions. Cell viability was assessed via luminescence detection of ATP using a GloMax Discover plate reader (Promega, Cat No. GM3000).

### RT-qPCR

Cell lysates were obtained using RNeasy Mini Kit RLT buffer (QIAGEN, Cat No. 74106), enriched with 10% β-mercaptoethanol (Millipore Sigma, Cat. No. 444203-250ML) as a reducing agent. RNA isolation from lysates was carried out using the RNeasy Mini Kit spin columns and buffers RWI and RPE, according to the manufacturer’s manual. All samples were normalized to 10 ng/µL of RNA using the NanoDrop One Spectrophotometer (Thermo Fisher, Cat No. ND-ONE-W). For intracellular viral RNA analysis, RT-qPCR reactions were performed using the RNA UltraSense One-Step Quantitative RT-PCR System (Thermo Fisher Scientific, Cat No. 11732-927) with custom primers (forward: AAAGGAACAATGGACTCTGGTCA; reverse: CACTTCTTACTACCATGTCCTCCAAT) and TaqMan TAMRA probe (Thermo Fisher, Cat No. 450025 6FAM-AAAGCTTTGATATCTCTCAGTGCCCCAA-TAMRA), targeting the RVFV M segment. For gene expression assays, normalized RNA samples were analyzed using the TaqMan RNA-to-CT 1-Step Kit (Thermo Fisher, Cat No. 4392938) and TaqMan probes specific to genes of interest: CXCL10 (Hs00171042_m1), IL1B (Hs01555410_m1), PTGS2 (Hs00153133_m1), NOS2 (Hs01075529_m1), IL-6 (Hs00174131_m1), IFNA1 (Hs03044218_g1), IFNB1 (Hs01077958_s1), RelA (Hs01042014_m1), TNF (Hs00174128_m1), 18S (Hs99999901_s1), MYC (Hs00153408_m1), AXIN2 (Hs00610344_m1), and CCND1 (Hs00765553_m1). Fold changes were determined using the delta-delta Ct method using 18S as the endogenous control.

### Immunofluorescence imaging

HSAEC cells were seeded in glass coverslips (Neuvitro, Cat No. GG-22-1.5-PLL) placed in six-well plates at a confluency of 1.5 × 10^5^ cells per slide 24 h prior to the start of the experiment. Cells were pre-treated, infected, and post-treated as previously described. At 24 h post-infection, the supernatants were removed; slides were washed twice with PBS and fixed in 4% neutral-buffered paraformaldehyde solution for 10 min at room temperature. After the fixation step, the cells were quickly washed twice in PBS, followed by two 5-min washes. Cells were permeabilized with 0.1% Triton X-100 (Sigma, Cat No. T8787-100ML) in PBS for 20 min at room temperature, and again washed as described. A blocking step was added for 1 h at room temperature using a blocking buffer containing 1% BSA, 0.3 M glycine, and 0.025% Triton X-100 in PBS. Incubation with primary antibodies was done overnight at 4°C in antibody-specific dilution. NF-κB p65 (Cell Signaling, Cat No. 8242S) and β-catenin (Cat No. MA1-300) antibodies were used at a 1:400 dilution. The following day, slides were quickly washed once in 0.025% Triton X-100 in PBS, followed by three 5-min washes. Secondary Alexa Fluor antibodies (568 and 488 goat anti-mouse) were added at a 1:500 dilution in blocking buffer for 1 h at room temperature and protected from light. After 1 h of incubation, slides were again washed in 0.025% Triton X-100 as described, followed by one PBS wash and left in PBS to avoid drying out. In the last 0.025% Triton X-100 wash, DAPI nuclear staining (4′,6-diamidino-2-phenylindole, 1:1,000) was added at a 1:1,000 dilution. To finalize, coverslips were mounted on glass slides (Fisher Brand, Cat No. 125493) using 10 µL of Fluoromount-G (Thermo Fisher, Cat No. 00-4958-02). For nuclear-to-cytoplasmic ratio quantifications, cell slides were imaged using a BioTek Cytation5 Cell Imaging Multimode Reader, with cell number and ratio calculations obtained using Gen5 software. For co-localization detection and Pearson’s coefficient calculation between NFκB p65 and β-catenin, slides were imaged using the Zeiss LSM 880 confocal laser scanning microscope with a water-immersion 60× objective. Overall fluorescence intensity of NF-κB p65 and β-catenin was obtained using ImageJ Fiji software.

### Post-infection viability assay

HSAECs and Huh7 cells were seeded in 96-well white wall plates at 2 × 10^4^ cells per well. The following day, cells were either mock- or RVFV rMP-12-infected at MOIs of 1 or 10 for 1 h. After 1-h infection, two PBS (Gibco, Cat No. 10010023) washes were performed, and 100 µL of the supplemented cell culture media was added back until the time points of treatment. At 16 or 24 h post-infection, mock and RVFV rMP-12 wells were treated with BTX-A51 (HSAECs 10 µM and Huh7 5 µM) or an equal volume of DMSO in control wells until the time point of collection. At 48 h post-infection, existing media were removed, and 100 µL of fresh media with an equal volume of CellTiter-Glo reagent (Promega, Cat No. G7571) was added to each well. Cell viability was measured following the manufacturer’s protocol using a GloMax Discover plate reader (Promega, Cat No. GM3000) for ATP luminescence detection levels.

### Statistics

Statistical analysis to determine experimental significance between conditions was performed using Student’s unpaired *t*-test or a two-way analysis of variance (ANOVA) to compare the means of test vs control data. Differences between test and control data were considered statistically significant if the *P*-value was equal to or below 0.05. Biological replicates were performed in a number of four, unless otherwise noted in figure legends.

## Data Availability

The key data that support the findings of this study are presented in the article and its supplemental material. Any other data not shared in this article will be made available upon reasonable request to the authors.

## SUPPLEMENTAL MATERIAL

Supplemental material jvi.01042-26-s0001.docx Fig. S1 to S4.

ASM does not own the copyrights to Supplemental Material that may be linked to, or accessed through, an article. The authors have granted ASM a non-exclusive, world-wide license to publish the Supplemental Material files. Please contact the corresponding author directly for reuse.
