# Supplementary material for: BTX-A51 treatment modulates host innate immune responses via NF-κB and β-catenin signaling during Rift Valley fever virus infection in vitro
Source: J Virol. 2026 Sep 9;100(10):e01042-26. doi: 10.1128/jvi.01042-26 (PMC13712988; doi:10.1128/jvi.01042-26)
Supplement: Supplementary file 1 [file jvi.01042-26-s0001.docx]

Supplemental figure 1

**A)**

**B)**

**C)**

**Fig. S1 – Assessment of BTX-A51 50% cytotoxicity concentration (CC_50_) in different cell types**. A) HSAECs, B) Huh7 cells, and C) SK-N-SH cells were assessed for viability following treatment with serial dilutions of BTX-A51. CC50 values were calculated using the GraphPad Prism CC50 nonlinear regression analysis. Data are presented as mean ± SD (n = 4). All three cell types maintained ≥80% viability at BTX-A51 concentrations ≤20 μM.

Supplemental figure 2

**A)**

**B)**

**C)**

**Mock**

**Mock**

**Fig. S2 – BTX-A51 treatment decreases phospho-GSK-3β levels.** HSAECs were pre-treated with either DMSO (control) or BTX-A51 (10 μM) for 1 hour, followed by mock or RVFV rMP-12 infection (MOI 0.1) for 1 hour. Cells were subsequently maintained under the same treatment conditions until collection at 24 hpi. Cell lysates were harvested in BLB for Western blot analysis of phospho-GSK-3α (Ser21) and phospho-GSK-3β (Ser9). Data are shown as a mean ± SD (*n*=3). Statistical analyses were performed using two-way ANOVA with Holm-Šídák's multiple comparisons test. The following comparisons were assessed: Mock DMSO vs. Mock BTX-A51, RVFV DMSO vs. RVFV BTX-A51, and Mock DMSO vs. RVFV DMSO. Only statistically significant comparisons are shown. *P < 0.05, **P < 0.01.

Supplemental figure 3

**Fig. S3 – Cycle threshold (Ct) values of 18S gene.** RT-qPCR quantification of the 18S gene was performed showing Ct values non-significantly altered in a comparison of all four experimental conditions. Data were analyzed using GraphPad Prism and are presented as mean ± SD (n = 4). Statistical analyses were performed using two-way ANOVA with Holm-Šídák's multiple comparisons test. The following comparisons were assessed: Mock DMSO vs. Mock BTX-A51, RVFV DMSO vs. RVFV BTX-A51, and Mock DMSO vs. RVFV DMSO. No statistically significant comparisons were found.

Supplemental figure 4

**Fig. S4 – BTX-A51 does not significantly alter cell count in treated cultures.** HSAECs were pre-treated with either BTX-A51 (10 μM) or DMSO (control) for 1 hour prior to mock or RVFV rMP-12 infection (MOI 0.1). Following infection, cells were maintained under the corresponding treatment conditions until 24 hours post-infection, at which time they were harvested by trypsinization (Trypsin-EDTA) and counted using the Trypan blue exclusion assay. Data were analyzed using GraphPad Prism and are presented as mean ± SD (n = 4). Statistical analyses were performed using two-way ANOVA with Holm-Šídák's multiple comparisons test. The following comparisons were assessed: Mock DMSO vs. Mock BTX-A51, RVFV DMSO vs. RVFV BTX-A51, and Mock DMSO vs. RVFV DMSO. No statistically significant comparisons were found.
